# Race and Ethnicity of Infants Enrolled in Neonatal Clinical Trials: A Systematic Review

**DOI:** 10.1001/jamanetworkopen.2023.48882

**Published:** 2023-12-21

**Authors:** Allison N. J. Lyle, Henna Shaikh, Ellie Oslin, Megan M. Gray, Elliott Mark Weiss

**Affiliations:** 1 Department of Pediatrics, University of Washington School of Medicine, Seattle; 2 Treuman Katz Center for Pediatric Bioethics and Palliative Care, Seattle Children’s Research Institute, Seattle, Washington

## Abstract

**Importance:**

Representativeness of populations within neonatal clinical trials is crucial to moving the field forward. Although racial and ethnic disparities in research inclusion are well documented in other fields, they are poorly described within neonatology.

**Objective:**

To describe the race and ethnicity of infants included in a sample of recent US neonatal clinical trials and the variability in this reporting.

**Evidence Review:**

A systematic search of US neonatal clinical trials entered into Cochrane CENTRAL 2017 to 2021 was conducted. Two individuals performed inclusion determination, data extraction, and quality assessment independently with discrepancies adjudicated by consensus.

**Findings:**

Of 120 studies with 14 479 participants that met the inclusion criteria, 75 (62.5%) included any participant race or ethnicity data. In the studies that reported race and ethnicity, the median (IQR) percentage of participants of each background were 0% (0%-1%) Asian, 26% (9%-42%) Black, 3% (0%-12%) Hispanic, 0% (0%-0%) Indigenous (eg, Alaska Native, American Indian, and Native Hawaiian), 0% (0%-0%) multiple races, 57% (30%-68%) White, and 7% (1%-21%) other race or ethnicity. Asian, Black, Hispanic, and Indigenous participants were underrepresented, while White participants were overrepresented compared with a reference sample of the US clinical neonatal intensive care unit (NICU) population from the Vermont Oxford Network. Many participants were labeled as other race or ethnicity without adequate description. There was substantial variability in terms and methods of reporting race and ethnicity data. Geographic representation was heavily skewed toward the Northeast, with nearly one-quarter of states unrepresented.

**Conclusions and Relevance:**

These findings suggest that neonatal research may perpetuate inequities by underrepresenting Asian, Black, Hispanic, and Indigenous neonates in clinical trials. Studies varied in documentation of race and ethnicity, and there was regional variation in the sites included. Based on these findings, funders and clinical trialists are advised to consider a 3-point targeted approach to address these issues: prioritize identifying ways to increase diversity in neonatal clinical trial participation, agree on a standardized method to report race and ethnicity among neonatal clinical trial participants, and prioritize the inclusion of participants from all regions of the US in neonatal clinical trials.

## Key Points

**Question:**

What are the races and ethnicities of participants in neonatal clinical trials and how are race and ethnicity reported?

**Findings:**

This systematic review of 120 studies with 14 479 participants found that Asian, Black, Hispanic, and Indigenous (eg, Alaska Native, American Indian, and Native Hawaiian) participants were underrepresented in neonatal clinical trials, while White participants were overrepresented. There was wide variation in the terms used to report race and ethnicity data, and geographic representation was unevenly distributed, with some central and western US regions underrepresented.

**Meaning:**

These findings suggest that inclusion of a more diverse population of participants within US neonatal clinical trials should be prioritized and that standardization is needed in reporting the race and ethnicity of participants in neonatal clinical trials.

## Teaser

This systematic review describes the race and ethnicity of infants included in a sample of recent US neonatal clinical trials and the variability in this reporting.

## Introduction

Neonatal research is the key mechanism to advance medical knowledge, improve treatment options, and optimize outcomes within neonatology. Including a diverse population in research studies is critical to improving patient outcomes. Difficulty recruiting from marginalized populations, such as Black communities, is a prevalent and long-standing problem in adult medical research.^1^ Underrepresentation of Black and Hispanic children has been documented in pediatric critical care and oncology research.^2,3,4^ Although data from neonatal research are limited, those available suggest similar underrepresentation. Within neonatal research, Black infants were less likely to enroll in Neonatal Research Network studies compared with Hispanic or White infants.^5^ Parents on Medicaid, those reporting lower income, and those identifying as Black were less likely to enroll their infant in a multicenter neonatal clinical trial.^6^

Failure to include minoritized racial and ethnic populations in neonatal clinical trials is particularly problematic because these populations have significantly worse clinical outcomes. Black mothers and birthing parents have higher preterm birth rates than their White counterparts, a gap that has increased over time.^7^ There are significant disparities in outcomes for minoritized infants, including both for term newborns^8^ and for preterm infants,^9,10,11^ resulting in increased morbidity, cost, and long-term disability.^12,13^ We must note that race is a social construct, and these disparities are due to systemic racism, not biological differences.^14,15^ Nonetheless, because of these documented worse outcomes, including minoritized infants in neonatal research is critical, as the underrepresentation of these populations in neonatal research threatens to exacerbate existing health disparities.

We sought to quantify the race and ethnicity of neonatal research participants and describe how race and ethnicity are reported. We included only clinical trials as they are the most fundamental contributors to the knowledge base. The primary objective was to identify the race and ethnicity of participants in neonatal clinical trials and compare with a reference population. We hypothesized that non-Hispanic and White infants would be overrepresented and that Hispanic infants and those with races other than White would be underrepresented. We compared these data with both US census data and data from the Vermont Oxford Network (VON) as the best estimates of available recent race and ethnicity data of the US neonatal intensive care unit (NICU) population. As a secondary objective, we aimed to describe the geographic location of sites participating in included studies.

## Methods

For this systematic review, we performed a systematic search of Cochrane CENTRAL in February 2022 with assistance from a research librarian (Table 1) following the Preferred Reporting Items for Systematic Reviews and Meta-analyses (PRISMA) reporting guidelines. We limited the search to full-length English-language articles on neonatal human studies conducted exclusively in the US and entered into Cochrane CENTRAL from 2017 to 2021. We included only peer-reviewed neonatal studies that met the National Institutes of Health definition of a clinical trial^16^ with at least 20 neonates. We excluded fetal studies, placental tissue studies, cord blood studies, well newborns, and infants not born in the US. To avoid the potential of participants in a study being represented multiple times, only the first published report from a clinical trial was eligible. Our planned methods were published on PROSPERO (record No. 240234).

**Table 1.** Search Criteria for Studies

Criterion	Search terms
1	(infant* or baby or babies or neonat* or “neo nate” or “neo nates” or “new born” or “newly born” or “new born” or newborn*)
2	(“intensive care” or “critical care” or ICU or ICUs or CICU or CICUs or “special care nursery” or “special care nurseries” or ((intensive* or critical*) NEAR/3 (therap* or treat* or care) NEAR/3 (unit or units or department* or ward or wards)) or (“close attention” NEAR/1 (unit or units or department* or ward or wards)))
3	[mh “intensive care units, neonatal”] OR [mh “intensive care, neonatal”]
4	NICU or NICUs
5	(1 AND 2) OR 3 OR 4
6	(1 AND 2) OR 3 OR 4; limits: publication year from 2017 to 2021, in trials

Abbreviations: ICU, intensive care unit; mh, medical subject heading; NICU, neonatal intensive care unit.

Our ideal comparison group would be all patients admitted to US NICUs. As no nationally available data on NICU admissions contain race and ethnicity data, we used a large VON publication on very preterm infants for this comparison.^17^ The major disadvantage of this data set was its focus solely on preterm infants. Its advantages were that it was recent (infants born 2014-2016) and captured 743 NICUs and 122 269 infants across all US regions. The VON data set included the percentages of preterm infants by race and ethnicity, including 4.8% Asian neonates, 29.7% Black neonates, 17.8% Hispanic neonates, 0.7% Indigenous (eg, Alaska Native, American Indian, and Native Hawaiian), 44.1% White neonates, and 2.8% neonates with race and ethnicity reported as other or unknown. US National Vital Statistics data on race and ethnicity were also reviewed and showed the racial composition of all live births in the US during 2018 to 2020 as 7% Asian neonates, 15% Black neonates, 24% Hispanic neonates, 0.8% Indigenous neonates, and 52% White neonates.^17^ The discrepancy between the race and ethnicity of preterm infants and all live-born infants is likely due to the disproportionate number of Black infants born preterm. Given the discrepancies in these 2 potential reference samples, we included both as comparisons to establish our best estimate of the US clinical NICU population.

Two researchers (including A.N.J.L., H.S., or E.O.) independently screened the abstracts and titles of articles for potential inclusion. The full text of the remaining articles was obtained and evaluated against the same inclusion and exclusion criteria. Disagreement on which articles met inclusion criteria was settled by consensus of 3 team members (including A.N.J.L., H.S., E.O., and E.M.W.). Data extraction included any available race and ethnicity data of participants and geographic locations of participating sites. Two team members (including A.N.J.L., H.S., or E.O.) performed data extraction independently. Discrepancies were settled by group consensus with at least 3 authors (A.N.J.L., H.S., E.O., and E.M.W.). The University of Washington REDCap database was used for data collection and management.^18,19^ Two independent researchers assessed the quality and potential for bias of each article using the scoring system for methodological rigor developed by Hawker et al,^20^ which prompts users to rank different domains and aspects of study reporting (eg, abstract and title, implications and usefulness) on a 4-point Likert scale, ranging from 4, indicating good to 1, very poor. Scores were then added up to determine the overall study quality, as others have done previously.^21,22^

### Statistical Analysis

Data were analyzed with Stata version 18 (StataCorp), and graphics were created using Tableau software version 2022 (Tableau Software). Descriptive statistics were used to tabulate counts and percentages. Percentages were reported for both percentages of individuals with a stated race or ethnicity per study and for the percentage of the whole pooled study sample. The comparison of reporting of race and ethnicity data over time was evaluated with 2-sided Fisher exact testing with significance set at *P* = .05. Data were analyzed in March 2023, with additional confirmatory analyses performed in October 2023.

## Results

We identified 120 studies^23,24,25,26,27,28,29,30,31,32,33,34,35,36,37,38,39,40,41,42,43,44,45,46,47,48,49,50,51,52,53,54,55,56,57,58,59,60,61,62,63,64,65,66,67,68,69,70,71,72,73,74,75,76,77,78,79,80,81,82,83,84,85,86,87,88,89,90,91,92,93,94,95,96,97,98,99,100,101,102,103,104,105,106,107,108,109,110,111,112,113,114,115,116,117,118,119,120,121,122,123,124,125,126,127,128,129,130,131,132,133,134,135,136,137,138,139,140,141,142^ that were entered into Cochrane CENTRAL 2017 to 2021 and met our inclusion criteria (eFigure 1 in Supplement 1). There was a range in the articles’ availability via electronic publication (2016-2021) and publication year (2017-2023). In total, 14 479 participants from study sites with 174 unique zip codes spanning 38 states and the District of Columbia were represented in these studies. The studies evaluated a wide range of interventions within neonatology, including procedural (eg, delayed cord clamping), pharmacologic (eg, antithrombin), feeding (eg, protein supplementation), and diagnostic (eg, genome sequencing) (eTable in Supplement 1).^23,24,25,26,27,28,29,30,31,32,33,34,35,36,37,38,39,40,41,42,43,44,45,46,47,48,49,50,51,52,53,54,55,56,57,58,59,60,61,62,63,64,65,66,67,68,69,70,71,72,73,74,75,76,77,78,79,80,81,82,83,84,85,86,87,88,89,90,91,92,93,94,95,96,97,98,99,100,101,102,103,104,105,106,107,108,109,110,111,112,113,114,115,116,117,118,119,120,121,122,123,124,125,126,127,128,129,130,131,132,133,134,135,136,137,138,139,140,141,142^

There were 75 studies^25,26,27,30,32,33,34,37,38,39,40,41,42,43,44,47,48,49,51,53,57,58,61,62,64,68,70,72,73,74,75,77,78,81,82,83,87,88,90,91,92,94,95,96,97,98,100,104,105,107,108,109,110,115,116,119,120,121,122,123,124,125,126,128,129,130,131,132,133,134,135,136,137,138,141^ (62.5%) that included participant race and ethnicity data of any kind (eTable in Supplement 1). Most studies reported race and ethnicity as a single combined variable (eg, non-Hispanic White), while some reported them as separate variables (eg, non-Hispanic and White).

Among the 75 studies^25,26,27,30,32,33,34,37,38,39,40,41,42,43,44,47,48,49,51,53,57,58,61,62,64,68,70,72,73,74,75,77,78,81,82,83,87,88,90,91,92,94,95,96,97,98,100,104,105,107,108,109,110,115,116,119,120,121,122,123,124,125,126,128,129,130,131,132,133,134,135,136,137,138,141^ that reported race and ethnicity, there was a pooled sample of 10 650 participants. This pooled sample was classified as 1.2% Asian participants, 33.0% Black participants, 11.2% Hispanic participants, 0.2% Indigenous, 0.8% participants identifying as multiple races, 49.3% White participants, and 10.3% participants identifying as other race or ethnicity (eFigure 2 in Supplement 1).

The median (IQR) percentage of participants of each background were 0% (0%-1%) Asian participants, 26% (9%-42%) Black participants, 3% (0%-12%) Hispanic participants, 0% (0%-0%) Indigenous participants, 0% (0%-0%) participants identifying as multiple races, 57% (30%-68%) White participants, and 7% (1%-21%) participants identifying as other race or ethnicity. The difference between aggregate and median percentage was particularly notable among Black participants, because 10 studies^38,49,77,97,110,120,121,122,131,133^ had very high percentages of Black participants. Among these studies with more than half of the sample reported as Black participants, 9 studies^38,49,97,110,120,121,122,131,133^ were single-centered trials in cities with a predominantly Black population and 1 study^77^ had Black race as an inclusion criterion.

We evaluated study methods to identify where race and ethnicity information originated and whom it described. A few studies specified the source of race and ethnicity information: 8 studies^32,77,82,88,90,91,96^ reported that it was from parental report and 1 study^92^ reported that it was from the medical record; the other 66 studies did not specify. Of studies that specified whose race was reported, 31 studies^26,30,37,47,48,49,51,53,57,58,61,68,75,78,91,92,100,104,107,108,110,121,123,129,131,133,134,135,137,138,141^ reported the infant’s information, 13 studies^32,39,42,43,81,82,83,88,96,115,125,126,128^ reported the mother’s information (using the label *mother* specifically), and 1 study^77^ reported the mother’s, father’s (using the label *father* specifically), and infant’s information.

**Figure 1.**
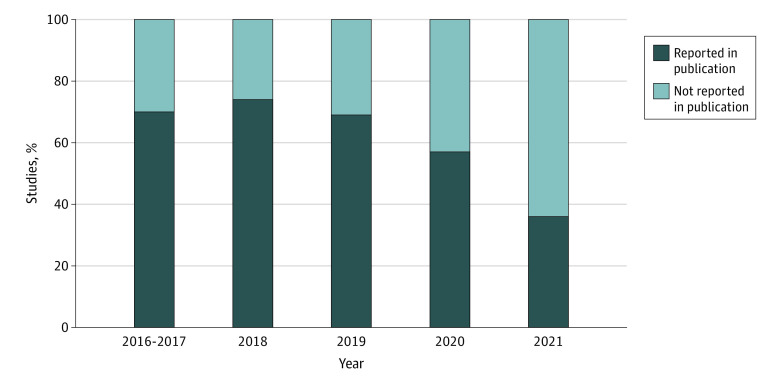
Reporting of Any Race or Ethnicity Data Over Time

The number of included studies each year was too low to meaningfully compare trends in reporting race and ethnicity data over time. However, exploring the binary outcome of reporting or not reporting race and ethnicity data was valuable. Figure 1 shows that the distribution of reporting vs not reporting race and ethnicity data in NICU clinical studies over the 5 years evaluated. We assessed reporting by NIH funding: of the 52 studies^23,24,28,29,30,36,37,38,43,44,47,50,57,59,63,64,67,69,70,74,75,77,78,82,86,87,88,90,91,94,95,96,99,105,107,109,110,115,118,119,120,121,122,125,126,127,132,135,137,139,140,142^ funded by the NIH, 35 studies^30,37,38,43,44,47,57,64,70,74,75,77,78,82,87,88,90,91,94,95,96,105,107,109,110,115,119,120,121,122,125,126,132,135,137^ (67%) included race and ethnicity data. Among 68 studies^14,26,27,31,32,33,34,35,39,40,41,42,45,46,48,49,51,52,53,54,55,56,58,60,61,62,65,66,68,71,72,73,76,79,80,81,83,84,85,89,92,93,97,98,100,101,102,103,104,106,108,111,112,113,114,116,117,123,124,125,128,129,130,131,133,134,136,138^ not funded by the NIH, 40 studies^14,26,27,32,33,34,39,40,41,42,48,49,51,53,58,61,62,68,72,73,81,83,92,97,98,100,104,108,116,123,124,125,128,129,130,131,133,134,136,138^ (58%) reported race and ethnicity data; the difference between NIH-funded and non-NIH–funded studies was not statistically significant by Pearson χ^2^.

**Figure 2.**
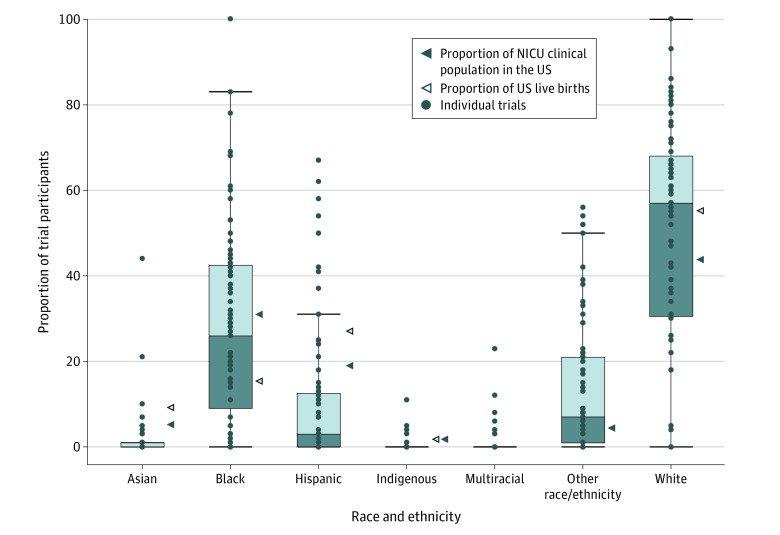
Race and Ethnicity of Participants Compared With the Best Estimate of the US Clinical Neonatal Intensive Care Unit (NICU) Population Dots indicate each individual trial’s proportion of participants in each racial and ethnic category; bars, medians; boxes, IQRs; whiskers, ranges.

We compared median percentages of reported race and ethnicity with the best available published clinical data on race and ethnicity in patients admitted to the NICU^143^ and with National Vital Statistics Data on US births.^17^ In our research population of 10 650 infants from 75 publications^25,26,27,30,32,33,34,37,38,39,40,41,42,43,44,47,48,49,51,53,57,58,61,62,64,68,70,72,73,74,75,77,78,81,82,83,87,88,90,91,92,94,95,96,97,98,100,104,105,107,108,109,110,115,116,119,120,121,122,123,124,125,126,128,129,130,131,132,133,134,135,136,137,138,141^ for whom any race and/or ethnicity data were available, Asian, Hispanic, and Indigenous participants were underrepresented compared with both comparator groups. Black infants (26% in our sample) were overrepresented compared with US birth statistics (15%) but underrepresented compared with the clinical NICU population (30%) (Figure 2). White participants were overrepresented (57%) compared with both comparator groups (52% of US live births, 44% of US clinical NICU population).

We evaluated the labels used by studies to describe the race and ethnicity of included infants. Studies used a range of labels to describe the race and ethnicity of participants (Table 2). For example, 8 labels were used by studies that reported any Black participants. In addition, there were many labels, typically poorly defined, to signify other, unknown, or not specified race and ethnicity (Table 2).

**Table 2.** Label Used to Describe Race and Ethnicity of Participants

Category	Label used^a^	No.	
		Studies using label	Infants categorized as label
Asian	Asian	19	102
	Asian American	1	2
	Asian or Pacific Islander	1	2
	Asian/Pacific Islander	1	17
Black	African American	21	672
	African American/Black	2	43
	African, African American	1	3
	Black	33	2439
	Black non-Hispanic	1	15
	Black or African American	3	76
	Non-Hispanic African American	1	34
	Non-Hispanic Black	1	232
Hispanic	Hispanic	32	1010
	Hispanic or Latino	6	112
	Hispanic/Latino	1	6
	Hispanic/Mexican	1	13
	Hispanic-Latino	1	9
	Latino	1	46
Indigenous	American Indian or Alaska Native	3	3
	American Indian/Alaskan Native	1	1
	Native American	2	5
	Native Hawaiian or other Pacific Islander	1	7
	Native Hawaiian or other Pacific Islander or other	1	2
Non-Hispanic	Non-Hispanic	2	144
	Non-Hispanic or Latino	1	71
	Not Hispanic	1	23
Multiple	Biracial	1	6
	More than 1	2	6
	Multiracial	3	64
	Multiple/biracial	1	5
White	Caucasian	14	498
	Non-Hispanic White	2	270
	White	46	4449
	White non-Hispanic	2	31
Other	Missing	2	32
	Non-White	1	14
	Not reported	1	8
	Not specified	1	23
	Other	27	365
	Other/unknown	2	42
	Unknown	6	56
	Unknown ethnicity	2	16
	Unknown, not reported	2	86
	Unknown/undetermined ethnicity	1	3
	Unreported	1	6

^a^ The exact label used by each article is presented.

**Figure 3.**
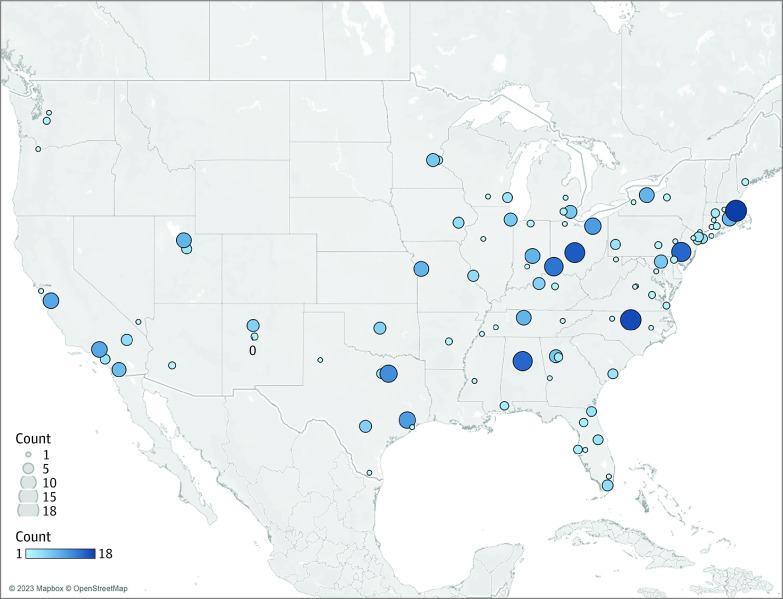
Map of Sites Contributing to Included Studies Map is based on longitude (generated) and latitude (generated). Color shows sum of counts. Details are shown for state and county. The view is filtered on inclusions (county and state), which includes 97 studies.

Finally, we also extracted data regarding the geographic location of every clinical site in each study, including hospital name, city, state, and zip code. In this set of 120 studies,^23,24,25,26,27,28,29,30,31,32,33,34,35,36,37,38,39,40,41,42,43,44,45,46,47,48,49,50,51,52,53,54,55,56,57,58,59,60,61,62,63,64,65,66,67,68,69,70,71,72,73,74,75,76,77,78,79,80,81,82,83,84,85,86,87,88,89,90,91,92,93,94,95,96,97,98,99,100,101,102,103,104,105,106,107,108,109,110,111,112,113,114,115,116,117,118,119,120,121,122,123,124,125,126,127,128,129,130,131,132,133,134,135,136,137,138,139,140,141,142^ there was regional variation in the study sites included: 38 states and the District of Columbia were represented (Figure 3). There was a skew to the Northeast US (40 studies^26,33,36,37,42,44,45,46,52,54,56,57,63,69,71,72,73,74,76,77,82,88,91,95,96,98,101,102,106,108,113,116,123,125,126,130,132,138,140,142^ [33.3%]). Study sites on the East Coast were more likely to contribute to multiple research studies.

## Discussion

The findings of this systematic review of data from 120 studies^23,24,25,26,27,28,29,30,31,32,33,34,35,36,37,38,39,40,41,42,43,44,45,46,47,48,49,50,51,52,53,54,55,56,57,58,59,60,61,62,63,64,65,66,67,68,69,70,71,72,73,74,75,76,77,78,79,80,81,82,83,84,85,86,87,88,89,90,91,92,93,94,95,96,97,98,99,100,101,102,103,104,105,106,107,108,109,110,111,112,113,114,115,116,117,118,119,120,121,122,123,124,125,126,127,128,129,130,131,132,133,134,135,136,137,138,139,140,141,142^ including a large sample of US neonatal clinical trial participants (14 479 participants) support 3 important conclusions. First, Asian, Black, Hispanic, and Indigenous infants were underrepresented in these studies compared with our proxy for the US clinical NICU population. Second, there was substantial variation in the documentation of race and ethnicity, which complicates the accurate interpretation of sampling and trial findings. Third, there was substantial regional variation in participation in neonatal clinical trials.

Neonatal research risks perpetuating inequities by underrepresenting non-White neonates in clinical trials. A recent American Academy of Pediatrics policy statement affirms that “Race is not a biological category that produces disparities due to genetic differences; rather, it is a social category that can have devastating biological consequences.”^14^ While reporting race and ethnicity has the potential for benefit, it can also worsen health disparities if used to support spurious connections of race and ethnicity with medical outcomes.^144^

### Underrepresentation of Minoritized Racial and Ethnic Populations

Clinical trials must strive to mirror the populations impacted by the studied conditions to ensure they align with the ethical principles of beneficence and justice.^145,146^ Failing to include a diverse population in neonatal clinical trials deprives neonatal research of valuable information and threatens to exacerbate existing disparities in care.^8,9,10,11,147^ Our finding that Asian, Black, Hispanic, and Indigenous infants were underrepresented compared with our best estimate of the national NICU clinical population is consistent with the limited available data from the neonatal literature.^5,6^ The proportions of these 4 minoritized groups in our review were higher than a review of pediatric trials,^148^ which may be due to the sample (published articles vs ClinicalTrials.gov) or discipline (neonatology vs pediatrics).

We must prioritize increasing diversity within neonatal clinical trials, as recommended in the recently proposed 10 steps for increasing representativeness in neonatal clinical trials.^149^ Such work includes upstream issues, such as when certain populations are not approached due to a perceived disinterest in research or unavailability for follow-up.^150^ Language differences may also be a reason for not approaching certain groups, and processes can be put in place to overcome this barrier, such as hiring interpreters or multilingual research staff. Parents may differently consider reasons for and against participation when deciding whether to participate in research.^22,151^ Certain burdens of participation may be an annoyance for well-resourced individuals but deeply problematic for individuals with fewer resources.^152^ Ways to improve the enrollment processes, support relationship building between researchers and parents of potential participants,^153^ and decrease the burdens of participation should be considered as key targets of future work to decrease disparities in participation.

### Variation in Documentation of Race and Ethnicity Data

Studies in this systematic review varied in their documentation of race and ethnicity in at least 4 distinct ways: categories included, specific labels used, 1-variable vs 2-variable reporting, and reporting of the data source. Many studies did not report any race and ethnicity data at all, which is consistent with prior reviews.^148,154^ Studies reporting race and/or ethnicity did not consistently use the same categories. For example, not all studies that reported race and ethnicity included a category for Asian participants. Considering this issue on a granular level, among studies with no reported Asian participants, there was rarely a way to differentiate among 4 potential realities: (1) parent was not asked about race; (2) parent declined to answer; (3) parent chose an other race or ethnicity category; and (4) parent did not fit into available choices. If researchers fail to ask race and ethnicity questions in a way that supports meaningful answers from all parents, our data on these issues will remain incomplete and unclear. Research participants may be more reluctant to answer race and ethnicity questions if they are part of certain marginalized groups^155^ or if the options presented do not reflect their lived experience.^156^

The labels used to describe each race and ethnicity category were inconsistent. The updated *AMA Manual of Style* emphasizes that “terminology, usage, and word choice are critically important, especially when describing people and discussing race and ethnicity.”^157^ Heterogeneity of the labels used makes it nearly impossible to compare studies meaningfully. For example, 5 different labels were used to describe Indigenous participants. This variation in labeling is important because respondents may respond differently to the presented labels and because the variation makes it more difficult to compare race and ethnicity data across studies.^156^ The heterogeneity of labels used diminishes data quality and decreases the ability of researchers to address disparities in research inclusion.^158,159^

The issue of 1-variable (eg, describing a participant as Hispanic Black) vs 2-variable (eg, describing a participant as Hispanic and as Black) does not have an easy solution. Most studies reported race and ethnicity as a single variable in our sample, consistent with prior work.^143,148^ However, racial and ethnic identity is deeply complex^160^ and seems unlikely to be perceived as 2 independent variables. The US census treats race and ethnicity as separate and independent constructs,^161^ further complicating comparisons with 1-variable data. Recent *JAMA* guidelines^162^ on the reporting of race and ethnicity in medical journals did not address the issue of 1-variable vs 2-variable reporting.

Our findings reflect that, both within medicine and more broadly, there is no consensus on how to query, report, and analyze data from multiracial individuals.^160^ None of the included studies explicitly discussed allowing individuals to choose more than 1 race category, and very few offered a multiple races option. Furthermore, the variability in other categories makes it difficult to compare between studies: for example, parents could sometimes select an other option, and sometimes other was used to describe nonrespondents. In the 2020 census, nearly 15% of individuals selected “two or more races,” indicating a growing need to ensure accurate reporting of multiracial individuals in research.^163^

Few studies reported the data source of race and ethnicity; this is important because there can be discrepancies between self-reported race and ethnicity and that documented in the medical record, particularly for the Hispanic population.^164^ In updated guidance for the *AMA Manual of Style*,^157^ identifying the source of race and ethnicity data (eg, self-report or electronic medical record) is recommended and consistent with recent recommendations for increasing representativeness in neonatal clinical trials.^149^

Researchers have increasingly understood that race is a social construct, and there is an evolving appreciation of the potential problems of attributing differences by race to genetics rather than as markers of systemic inequities.^14,15^ It would be deeply disappointing if researchers, being unsure how to work with these data, did not present them at all. A review of pediatric trials from 2008 to 2018 showed an increase in reporting of race and ethnicity over time.^148^

Researchers, funders, and journal editors must agree on a standard method to ask, report, and analyze the race and ethnicity of neonatal clinical trial participants. Issues to address include separate or combined race and ethnicity variables, data origination, standardization of labels, categorization of multiracial infants, and the use of an other category. The interplay of race and ethnicity is a deeply complicated issue,^160^ and guidelines will need to be changed over time as they are used and as best practices evolve. Starting with established general guidelines^162^ and adjusting to the neonatal clinical trial context could be a good start. Simply requiring that all published neonatal clinical trials report any race and ethnicity data would be a huge step in the right direction.

### Regional Variation in Participation in Neonatal Clinical Trials

There was geographic variability of included sites, with an overrepresentation of infants from the eastern portion of the US. Large portions of the country with minimal or no representation (eg, the American West and South) in this group of published trials included much of the rural, Asian,^166^ and Indigenous^165^ populations in the US. An Australian study^167^ also has noted the underrepresentation of the rural patient population in clinical research and the need to focus on increasing research capacity in such areas.

Broader geographic inclusion of infants within neonatal clinical trials should be prioritized. US regions with the highest populations of the most underrepresented populations, particularly Asian, Hispanic, and Indigenous infants,^143,168,169^ should be prioritized. Increased inclusion of sites within the western US could be an effective way to narrow the gap between the clinical and research populations.

### Limitations

This study has some limitations. In an attempt to capture all US-based neonatal clinical trials, we used a variety of search terms, chosen with the assistance of an experienced research librarian; however, it is possible that we missed relevant articles. We excluded articles including infants from both the US and other countries. This was necessary because country-level demographic data were never available, but it limits the generalizability of our findings. If more granular data were available, future work should ideally include clinical trials that include both US and international NICU patients.

## Conclusions

This systematic review of recently published US neonatal clinical trials found that Asian, Black, Hispanic, and Indigenous infants were underrepresented. Studies varied in whether and how race and ethnicity data were documented. There was regional variation in the sites included. Based on these findings, we propose the following recommendations: (1) prioritize increasing diversity within neonatal clinical trials, including research that aims to identify empirically sound and ethically acceptable ways to do so; (2) agree on a standardized method to ask, report, and analyze race and ethnicity of neonatal clinical trial participants; and (3) prioritize the inclusion of participants from all US regions.

## Notes

Supplement 1. **eTable.** Characteristics of Included Studies **eFigure 1.** Flowchart of Included Studies **eFigure 2.** Race and Ethnicity of Participants
eReferences.

Supplement 2.
Data Sharing Statement

## References

[1] Paskett ED, Reeves KW, McLaughlin JM, . Recruitment of minority and underserved populations in the United States: the Centers for Population Health and Health Disparities experience. Contemp Clin Trials. 2008;29(6):847-861. doi:10.1016/j.cct.2008.07.006 18721901 PMC2642621

[2] Natale JE, Lebet R, Joseph JG, ; Randomized Evaluation of Sedation Titration for Respiratory Failure (RESTORE) Study Investigators. Racial and ethnic disparities in parental refusal of consent in a large, multisite pediatric critical care clinical trial. J Pediatr. 2017;184:204-208.e1. doi:10.1016/j.jpeds.2017.02.006 28410087

[3] Paquette E, Shukla A, Duyar S. Social Determinants of Research Engagement and Implications for Precision Medicine: Sociodemographic Factors Associated with Differential Enrollment in a Pediatric Critical Care Biorepository. Pediatric Academic Societies; 2018.

[4] Liu L, Krailo M, Reaman GH, Bernstein L; Surveillance, Epidemiology and End Results Childhood Cancer Linkage Group. Childhood cancer patients’ access to cooperative group cancer programs: a population-based study. Cancer. 2003;97(5):1339-1345. doi:10.1002/cncr.11192 12599243

[5] Foglia EE, Nolen TL, DeMauro SB, ; Eunice Kennedy Shriver National Institute of Child Health and Human Development Neonatal Research Network. Short-term outcomes of infants enrolled in randomized clinical trials vs those eligible but not enrolled. JAMA. 2015;313(23):2377-2379. doi:10.1001/jama.2015.5734 26080344 PMC4720434

[6] Weiss EM, Olszewski AE, Guttmann KF, . Parental factors associated with the decision to participate in a neonatal clinical trial. JAMA Netw Open. 2021;4(1):e2032106. doi:10.1001/jamanetworkopen.2020.32106 33433595 PMC7804922

[7] Murphy SL, Xu J, Kochanek KD, Curtin SC, Arias E. Deaths: final data for 2015. Natl Vital Stat Rep. 2017;66(6):1-75. 29235985

[8] Glazer KB, Zeitlin J, Egorova NN, . Hospital quality of care and racial and ethnic disparities in unexpected newborn complications. Pediatrics. 2021;148(3):e2020024091. doi:10.1542/peds.2020-024091 34429339 PMC9708325

[9] Anderson JG, Rogers EE, Baer RJ, . Racial and ethnic disparities in preterm infant mortality and severe morbidity: a population-based study. Neonatology. 2018;113(1):44-54. doi:10.1159/000480536 29073624

[10] Wallace ME, Mendola P, Kim SS, . Racial/ethnic differences in preterm perinatal outcomes. Am J Obstet Gynecol. 2017;216(3):306.e1-306.e12. doi:10.1016/j.ajog.2016.11.1026 27865977 PMC5572800

[11] Karvonen KL, Baer RJ, Rogers EE, . Racial and ethnic disparities in outcomes through 1 year of life in infants born prematurely: a population based study in California. J Perinatol. 2021;41(2):220-231. doi:10.1038/s41372-021-00919-9 33514879

[12] Beck AF, Edwards EM, Horbar JD, Howell EA, McCormick MC, Pursley DM. The color of health: how racism, segregation, and inequality affect the health and well-being of preterm infants and their families. Pediatr Res. 2020;87(2):227-234. doi:10.1038/s41390-019-0513-6 31357209 PMC6960093

[13] Janevic T, Zeitlin J, Auger N, . Association of race/ethnicity with very preterm neonatal morbidities. JAMA Pediatr. 2018;172(11):1061-1069. doi:10.1001/jamapediatrics.2018.2029 30208467 PMC6248139

[14] American Academy of Pediatrics Board of Directors and Executive Committee. AAP perspective: race-based medicine. Pediatrics. 2021;148(4):e2021053829. doi:10.1542/peds.2021-053829 34353893

[15] Lett E, Asabor E, Beltrán S, Cannon AM, Arah OA. Conceptualizing, contextualizing, and operationalizing race in quantitative health sciences research. Ann Fam Med. 2022;20(2):157-163. doi:10.1370/afm.2792 35045967 PMC8959750

[16] National Institutes of Health. NIH’s definition of a clinical trial. Accessed November 2, 2021. https://grants.nih.gov/policy/clinical-trials/definition.htm

[17] Centers for Disease Control and Prevention. National Vital Statistics System: birth data. Accessed June 29, 2023. https://www.cdc.gov/nchs/nvss/births.htm

[18] Harris PA, Taylor R, Minor BL, ; REDCap Consortium. The REDCap consortium: building an international community of software platform partners. J Biomed Inform. 2019;95:103208. doi:10.1016/j.jbi.2019.103208 31078660 PMC7254481

[19] Harris PA, Taylor R, Thielke R, Payne J, Gonzalez N, Conde JG. Research electronic data capture (REDCap)—a metadata-driven methodology and workflow process for providing translational research informatics support. J Biomed Inform. 2009;42(2):377-381. doi:10.1016/j.jbi.2008.08.010 18929686 PMC2700030

[20] Hawker S, Payne S, Kerr C, Hardey M, Powell J. Appraising the evidence: reviewing disparate data systematically. Qual Health Res. 2002;12(9):1284-1299. doi:10.1177/1049732302238251 12448672

[21] Cavolo A, Dierckx de Casterlé B, Naulaers G, Gastmans C. Physicians’ attitudes on resuscitation of extremely premature infants: a systematic review. Pediatrics. 2019;143(6):e20183972. doi:10.1542/peds.2018-3972 31076541

[22] Nathe JM, Oskoui TT, Weiss EM. Parental views of facilitators and barriers to research participation: systematic review. Pediatrics. 2023;151(1):e2022058067. doi:10.1542/peds.2022-058067 36477217 PMC9808610

[23] Abbey NV, Mashruwala V, Weydig HM, . Electrocardiogram for heart rate evaluation during preterm resuscitation at birth: a randomized trial. Pediatr Res. 2022;91(6):1445-1451. doi:10.1038/s41390-021-01731-z 34645954 PMC8513736

[24] Alrifai MW, Mulherin DP, Weinberg ST, Wang L, Lehmann CU. Parenteral protein decision support system improves protein delivery in preterm infants: a randomized clinical trial. JPEN J Parenter Enteral Nutr. 2018;42(1):219-224. doi:10.1002/jpen.1034 29505147 PMC5841609

[25] Amaro CM, Bello JA, Jain D, . Early caffeine and weaning from mechanical ventilation in preterm infants: a randomized, placebo-controlled trial. J Pediatr. 2018;196:52-57. doi:10.1016/j.jpeds.2018.01.010 29519541

[26] Amatya S, Macomber M, Bhutada A, Rastogi D, Rastogi S. Sudden versus gradual pressure wean from Nasal CPAP in preterm infants: a randomized controlled trial. J Perinatol. 2017;37(6):662-667. doi:10.1038/jp.2017.10 28230835 PMC5446290

[27] Anderson-Berry A, Thoene M, Wagner J, . Randomized trial of two doses of vitamin D_3_ in preterm infants <32 weeks: dose impact on achieving desired serum 25(OH)D_3_ in a NICU population. PLoS One. 2017;12(10):e0185950. doi:10.1371/journal.pone.0185950 29016653 PMC5634602

[28] Angeles DM, Boskovic DS, Tan JC, . Oral dextrose reduced procedural pain without altering cellular ATP metabolism in preterm neonates: a prospective randomized trial. J Perinatol. 2020;40(6):888-895. doi:10.1038/s41372-020-0634-0 32103160 PMC7253349

[29] Angeles DM, Boskovic DS, Deming D, . A pilot study on the biochemical effects of repeated administration of 24% oral sucrose vs 30% oral dextrose on urinary markers of adenosine triphosphate degradation. J Perinatol. 2021;41(12):2761-2765. doi:10.1038/s41372-021-01239-8 34671099

[30] Arnold C, Tyson JE, Pedroza C, . Cycled phototherapy dose-finding study for extremely low-birth-weight infants: a randomized clinical trial. JAMA Pediatr. 2020;174(7):649-656. doi:10.1001/jamapediatrics.2020.0559 32338720 PMC7186919

[31] Atchley CB, Cloud A, Thompson D, . Enhanced protein diet for preterm infants: a prospective, randomized, double-blind, controlled trial. J Pediatr Gastroenterol Nutr. 2019;69(2):218-223. doi:10.1097/MPG.0000000000002376 31058772

[32] Backes CH, Cooper JN, Notestine JL, . A trial comparing continuous positive airway pressure (CPAP) devices in preterm infants. J Perinatol. 2020;40(8):1193-1201. doi:10.1038/s41372-020-0690-5 32433510 PMC7375950

[33] Balakrishnan M, Jennings A, Przystac L, . Growth and neurodevelopmental outcomes of early, high-dose parenteral amino acid intake in very low birth weight infants: a randomized controlled trial. JPEN J Parenter Enteral Nutr. 2018;42(3):597-606. doi:10.1177/0148607117696330 29187120

[34] Ballengee CR, Davalian F, Conaway MR, Sauer CG, Kaufman DA. Erythromycin and reflux events in premature neonates: a randomized clinical trial. J Pediatr Gastroenterol Nutr. 2018;67(6):720-725. doi:10.1097/MPG.0000000000002086 29985874

[35] Bedwell SM, Buster B, Sekar K. The effect of a continuous milk warming system on weight gain in very low birth-weight infants: a randomized controlled trial. Adv Neonatal Care. 2021;21(4):E86-E92. doi:10.1097/ANC.0000000000000818 33427754

[36] Blakely ML, Tyson JE, Lally KP, ; Eunice Kennedy Shriver National Institute of Child Health, Human Development Neonatal Research Network. Initial laparotomy versus peritoneal drainage in extremely low birthweight infants with surgical necrotizing enterocolitis or isolated intestinal perforation: a multicenter randomized clinical trial. Ann Surg. 2021;274(4):e370-e380. doi:10.1097/SLA.0000000000005099 34506326 PMC8439547

[37] Bogen DL, Hanusa BH, Baker R, Medoff-Cooper B, Cohlan B. Randomized clinical trial of standard- versus high-calorie formula for methadone-exposed infants: a feasibility study. Hosp Pediatr. 2018;8(1):7-14. doi:10.1542/hpeds.2017-0114 29263124 PMC5744267

[38] Brandon DH, Silva SG, Park J, Malcolm W, Kamhawy H, Holditch-Davis D. Timing for the introduction of cycled light for extremely preterm infants: a randomized controlled trial. Res Nurs Health. 2017;40(4):294-310. doi:10.1002/nur.21797 28431191 PMC5522348

[39] Brion LP, Rosenfeld CR, Heyne R, . Optimizing individual nutrition in preterm very low birth weight infants: double-blinded randomized controlled trial. J Perinatol. 2020;40(4):655-665. doi:10.1038/s41372-020-0609-1 32071367

[40] Brusseau C, Burnette T, Heidel RE. Clonidine versus phenobarbital as adjunctive therapy for neonatal abstinence syndrome. J Perinatol. 2020;40(7):1050-1055. doi:10.1038/s41372-020-0685-2 32424335

[41] Bruzoni M, Jaramillo JD, Dunlap JL, . Sutureless vs sutured gastroschisis closure: a prospective randomized controlled trial. J Am Coll Surg. 2017;224(6):1091-1096.e1. doi:10.1016/j.jamcollsurg.2017.02.014 28279777

[42] Butler-O’Hara M, Reininger A, Wang H, Amin SB, Rodgers NJ, D’Angio CT. A randomized controlled trial of glycerin suppositories during phototherapy in premature neonates. J Obstet Gynecol Neonatal Nurs. 2017;46(2):220-228. doi:10.1016/j.jogn.2016.10.008 28082211

[43] Calkins KL, Havranek T, Kelley-Quon LI, . Low-dose parenteral soybean oil for the prevention of parenteral nutrition-associated liver disease in neonates with gastrointestinal disorders. JPEN J Parenter Enteral Nutr. 2017;41(3):404-411. doi:10.1177/0148607115588334 26024828 PMC4663189

[44] Ceyhan-Birsoy O, Murry JB, Machini K, ; BabySeq Project Team. Interpretation of genomic sequencing results in healthy and ill newborns: results from the BabySeq Project. Am J Hum Genet. 2019;104(1):76-93. doi:10.1016/j.ajhg.2018.11.016 30609409 PMC6323417

[45] Choi PS, Emani S, Ibla JC, Marturano JE, Lowery TJ, Emani S. Magnetic resonance-based diagnostics for bleeding assessment in neonatal cardiac surgery. Ann Thorac Surg. 2020;109(6):1931-1936. doi:10.1016/j.athoracsur.2019.11.010 31887277

[46] Cholette JM, Swartz MF, Rubenstein J, . Outcomes using a conservative versus liberal red blood cell transfusion strategy in infants requiring cardiac operation. Ann Thorac Surg. 2017;103(1):206-214. doi:10.1016/j.athoracsur.2016.05.049 27496630

[47] Chorna OD, L Hamm E, Shrivastava H, Maitre NL. Feasibility of event-related potential (ERP) biomarker use to study effects of mother’s voice exposure on speech sound differentiation of preterm infants. Dev Neuropsychol. 2018;43(2):123-134. doi:10.1080/87565641.2018.1433671 29436854 PMC6141308

[48] Claassen CC, Hillman NH, Brown K, Williams HL, Strand ML. Comparison of bubble CPAP devices using RAM cannula for extubation failure in very low birth weight infants: randomized and cohort studies. Neonatology. 2019;115(1):28-35. doi:10.1159/000493156 30278459

[49] Cook LM, Nichols-Dada J, Damani S, . Randomized clinical trial of 24% oral sucrose to decrease pain associated with peripheral intravenous catheter insertion in preterm and term newborns. Adv Neonatal Care. 2017;17(1):E3-E11. doi:10.1097/ANC.0000000000000326 27533333

[50] Cooper DM, Girolami GL, Kepes B, . Body composition and neuromotor development in the year after NICU discharge in premature infants. Pediatr Res. 2020;88(3):459-465. doi:10.1038/s41390-020-0756-2 31926484 PMC7351612

[51] Corrigan MJ, Keeler JR, Miller HD, Ben Khallouq BA, Fowler SB. Music therapy and retinopathy of prematurity screening: using recorded maternal singing and heartbeat for post exam recovery. J Perinatol. 2020;40(12):1780-1788. doi:10.1038/s41372-020-0719-9 32681063

[52] Cummings JJ, Gerday E, Minton S, ; AERO-02 STUDY INVESTIGATORS. Aerosolized calfactant for newborns with respiratory distress: a randomized trial. Pediatrics. 2020;146(5):e20193967. doi:10.1542/peds.2019-3967 33060258

[53] Czarnecki ML, Hainsworth K, Simpson PM, ; Continuous Opioid Infusion in the Neonatal Intensive Care Unit. A pilot randomized controlled trial of outcomes associated with parent-nurse controlled analgesia vs continuous opioid infusion in the neonatal intensive care unit. Pain Manag Nurs. 2020;21(1):72-80. doi:10.1016/j.pmn.2019.08.002 31494028 PMC6980456

[54] Czynski AJ, Davis JM, Dansereau LM, . Neurodevelopmental outcomes of neonates randomized to morphine or methadone for treatment of neonatal abstinence syndrome. J Pediatr. 2020;219:146-151.e1. doi:10.1016/j.jpeds.2019.12.018 31987653 PMC7161698

[55] Daniel JM, Davidson LN, Havens JR, Bauer JA, Shook LA. Aromatherapy as an adjunctive therapy for neonatal abstinence syndrome: a pilot study. J Opioid Manag. 2020;16(2):119-125. doi:10.5055/jom.2020.0558 32329887

[56] Davidson J, Ruthazer R, Maron JL. Optimal timing to utilize olfactory stimulation with maternal breast milk to improve oral feeding skills in the premature newborn. Breastfeed Med. 2019;14(4):230-235. doi:10.1089/bfm.2018.0180 30882237 PMC10027347

[57] Davis JM, Shenberger J, Terrin N, . Comparison of safety and efficacy of methadone vs morphine for treatment of neonatal abstinence syndrome: a randomized clinical trial. JAMA Pediatr. 2018;172(8):741-748. doi:10.1001/jamapediatrics.2018.1307 29913015 PMC6142918

[58] Detmer MR, Evans K, Shina E, Walker K, DeLoach D, Malowitz JR. Multimodal neurologic enhancement improves preterm infants’ developmental outcomes: a longitudinal pilot study. Neonatal Netw. 2020;39(1):16-23. doi:10.1891/0730-0832.39.1.16 31919289

[59] DuPont TL, Baserga M, Lowe J, Zamora T, Beauman S, Ohls RK. Darbepoetin as a neuroprotective agent in mild neonatal encephalopathy: a randomized, placebo-controlled, feasibility trial. J Perinatol. 2021;41(6):1339-1346. doi:10.1038/s41372-021-01081-y 33986477 PMC8238874

[60] Elzein C, Urbas C, Hughes B, . Efficacy of nitric oxide administration in attenuating ischemia/reperfusion injury during neonatal cardiopulmonary bypass. World J Pediatr Congenit Heart Surg. 2020;11(4):417-423. doi:10.1177/2150135120911034 32645771

[61] Emery L, Hamm EL, Hague K, Chorna OD, Moore-Clingenpeel M, Maitre NL. A randomised controlled trial of protocolised music therapy demonstrates developmental milestone acquisition in hospitalised infants. Acta Paediatr. 2019;108(5):828-834. doi:10.1111/apa.14628 30375661

[62] Eze N, Murphy D, Dhar V, Rehan VK. Comparison of sprinting vs non-sprinting to wean nasal continuous positive airway pressure off in very preterm infants. J Perinatol. 2018;38(2):164-168. doi:10.1038/jp.2017.161 29072676

[63] Foglia EE, Ades A, Hedrick HL, . Initiating resuscitation before umbilical cord clamping in infants with congenital diaphragmatic hernia: a pilot feasibility trial. Arch Dis Child Fetal Neonatal Ed. 2020;105(3):322-326. doi:10.1136/archdischild-2019-317477 31462406 PMC7047568

[64] Forde D, Deming DD, Tan JC, . Oxidative stress biomarker decreased in preterm neonates treated with kangaroo mother care. Biol Res Nurs. 2020;22(2):188-196. doi:10.1177/1099800419900231 31973579 PMC7273802

[65] Frost BL, Patel AL, Robinson DT, Berseth CL, Cooper T, Caplan M. Randomized controlled trial of early docosahexaenoic acid and arachidonic acid enteral supplementation in very low birth weight infants. J Pediatr. 2021;232:23-30.e1. doi:10.1016/j.jpeds.2020.12.037 33358843

[66] Gautam NK, Pierre J, Edmonds K, . Transfusing platelets during bypass rewarming in neonates improves postoperative outcomes: a randomized controlled trial. World J Pediatr Congenit Heart Surg. 2020;11(1):71-76. doi:10.1177/2150135119888155 31835978

[67] Gaynor JW, Nicolson SC, Spray DM, . Remote ischemic preconditioning does not prevent white matter injury in neonates. Ann Thorac Surg. 2018;106(1):151-155. doi:10.1016/j.athoracsur.2018.02.060 29601806

[68] Gerges A, Gelfer P, Kennedy K. Randomized trial of earlier versus later oral feeding in very premature infants. J Perinatol. 2018;38(6):687-692. doi:10.1038/s41372-018-0058-2 29453433

[69] Glass KM, Greecher CP, Doheny KK. Oropharyngeal administration of colostrum increases salivary secretory iga levels in very low-birth-weight infants. Am J Perinatol. 2017;34(14):1389-1395. doi:10.1055/s-0037-1603655 28575910 PMC5685905

[70] Graham EM, Martin RH, Buckley JR, . Corticosteroid therapy in neonates undergoing cardiopulmonary bypass: randomized controlled trial. J Am Coll Cardiol. 2019;74(5):659-668. doi:10.1016/j.jacc.2019.05.060 31370958 PMC6684326

[71] Gray MM, Medoff-Cooper B, Enlow EM, Mukhopadhyay S, DeMauro SB. Every three-hour versus every six-hour oral feeding in preterm infants: a randomised clinical trial. Acta Paediatr. 2017;106(2):236-241. doi:10.1111/apa.13658 27862264

[72] Gupta K, Wang H, Amin SB. Soybean-oil lipid minimization for prevention of intestinal failure-associated liver disease in late-preterm and term infants with gastrointestinal surgical disorders. JPEN J Parenter Enteral Nutr. 2021;45(6):1239-1248. doi:10.1002/jpen.2004 32854150

[73] Hammer GB, Maxwell LG, Taicher BM, . Randomized population pharmacokinetic analysis and safety of intravenous acetaminophen for acute postoperative pain in neonates and infants. J Clin Pharmacol. 2020;60(1):16-27. doi:10.1002/jcph.1508 31448420 PMC6973014

[74] Hammond J, Kamboj R, Kashyap S, Sahni R. The interaction between diet and neurobehavior in very low birth weight infants. Pediatr Res. 2022;91(3):646-651. doi:10.1038/s41390-021-01464-z 33767376 PMC8463624

[75] Hart CK, Tawfik KO, Meinzen-Derr J, . A randomized controlled trial of Velcro versus standard twill ties following pediatric tracheotomy. Laryngoscope. 2017;127(9):1996-2001. doi:10.1002/lary.26608 28480522

[76] Havranek T, Shatzkin E, Chuang M, Xie X, Kim M, Rosen O. Respiratory outcomes after neonatal prone versus supine positioning following scheduled cesarean delivery: a randomized trial. J Matern Fetal Neonatal Med. 2021;34(18):2938-2944. doi:10.1080/14767058.2019.1674805 31564177

[77] Hibbs AM, Ross K, Kerns LA, . Effect of vitamin D supplementation on recurrent wheezing in Black infants who were born preterm: the D-Wheeze Randomized Clinical Trial. JAMA. 2018;319(20):2086-2094. doi:10.1001/jama.2018.5729 29800180 PMC6583240

[78] Jadcherla SR, Hasenstab KA, Wei L, . Role of feeding strategy bundle with acid-suppressive therapy in infants with esophageal acid reflux exposure: a randomized controlled trial. Pediatr Res. 2021;89(3):645-652. doi:10.1038/s41390-020-0932-4 32380509 PMC7647955

[79] Jakubowicz JF, Bai S, Matlock DN, . Effect of transcutaneous electrode temperature on accuracy and precision of carbon dioxide and oxygen measurements in the preterm infants. Respir Care. 2018;63(7):900-906. doi:10.4187/respcare.05887 29717098

[80] Jooste EH, Scholl R, Wu YH, . Double-blind, randomized, placebo-controlled trial comparing the effects of antithrombin versus placebo on the coagulation system in infants with low antithrombin undergoing congenital cardiac surgery. J Cardiothorac Vasc Anesth. 2019;33(2):396-402. doi:10.1053/j.jvca.2018.05.052 30072263

[81] Josephsen JB, Potter S, Armbrecht ES, Al-Hosni M. Umbilical cord milking in extremely preterm infants: a randomized controlled trial comparing cord milking with immediate cord clamping. Am J Perinatol. 2022;39(4):436-443. doi:10.1055/s-0040-1716484 32894871

[82] Juul SE, Comstock BA, Wadhawan R, ; PENUT Trial Consortium. A randomized trial of erythropoietin for neuroprotection in preterm infants. N Engl J Med. 2020;382(3):233-243. doi:10.1056/NEJMoa1907423 31940698 PMC7060076

[83] Kakkilaya V, Tang A, Wagner S, . Discontinuing nasal continuous positive airway pressure in infants ≤32 weeks of gestational age: a randomized control trial. J Pediatr. 2021;230:93-99.e3. doi:10.1016/j.jpeds.2020.10.045 33127365

[84] Katheria AC, Morales A, Shashank S, Rich WD, Finer NN. A pilot randomized trial of heart rate monitoring using conventional versus a new electrocardiogram algorithm during neonatal resuscitation at birth. J Pediatr. 2022;242:245-247.e1. doi:10.1016/j.jpeds.2021.10.037 34715091

[85] Katheria A, Arnell K, Brown M, . A pilot randomized controlled trial of EKG for neonatal resuscitation. PLoS One. 2017;12(11):e0187730. doi:10.1371/journal.pone.0187730 29099872 PMC5669495

[86] Kim CS, Grady N, Derrick M, . Effect of antibiotic use within first 48 hours of life on the preterm infant microbiome: a randomized clinical trial. JAMA Pediatr. 2021;175(3):303-305. doi:10.1001/jamapediatrics.2020.4916 33196773 PMC7670395

[87] Kingsmore SF, Cakici JA, Clark MM, ; RCIGM Investigators. A randomized, controlled trial of the analytic and diagnostic performance of singleton and trio, rapid genome and exome sequencing in ill infants. Am J Hum Genet. 2019;105(4):719-733. doi:10.1016/j.ajhg.2019.08.009 31564432 PMC6817534

[88] Kirpalani H, Bell EF, Hintz SR, ; Eunice Kennedy Shriver NICHD Neonatal Research Network. Higher or lower hemoglobin transfusion thresholds for preterm infants. N Engl J Med. 2020;383(27):2639-2651. doi:10.1056/NEJMoa2020248 33382931 PMC8487591

[89] Kochan M, Leonardi B, Firestine A, . Elevated midline head positioning of extremely low birth weight infants: effects on cardiopulmonary function and the incidence of periventricular-intraventricular hemorrhage. J Perinatol. 2019;39(1):54-62. doi:10.1038/s41372-018-0261-1 30348960

[90] Kotloff KL, Shirley DT, Creech CB, . Mupirocin for *Staphylococcus aureus* decolonization of infants in neonatal intensive care units. Pediatrics. 2019;143(1):e20181565. doi:10.1542/peds.2018-1565 30587533 PMC6317770

[91] Kraft WK, Adeniyi-Jones SC, Chervoneva I, . Buprenorphine for the treatment of the neonatal abstinence syndrome. N Engl J Med. 2017;376(24):2341-2348. doi:10.1056/NEJMoa1614835 28468518 PMC5662132

[92] Kumar N, Monga R, Sampath V, Ehrhart B. Prospective comparison of Enfamil and Similac liquid human milk fortifier on clinical outcomes in premature infants. Am J Perinatol. 2017;34(14):1411-1416. doi:10.1055/s-0037-1603940 28637062

[93] Lafferty MA, Mackley A, Green P, Ottenthal D, Locke R, Guillen U. Can Mozart improve weight gain and development of feeding skills in premature infants: a randomized trial. Am J Perinatol. 2023;40(7):793-798. doi:10.1055/s-0041-1731279 34157772

[94] Lam R, Schilling D, Scottoline B, . The effect of extended continuous positive airway pressure on changes in lung volumes in stable premature infants: a randomized controlled trial. J Pediatr. 2020;217:66-72.e1. doi:10.1016/j.jpeds.2019.07.074 31519441 PMC7986570

[95] Lambert LM, Trachtenberg FL, Pemberton VL, ; Pediatric Heart Network Investigators. Passive range of motion exercise to enhance growth in infants following the Norwood procedure: a safety and feasibility trial. Cardiol Young. 2017;27(7):1361-1368. doi:10.1017/S1047951117000427 28330522 PMC5712224

[96] Laptook AR, Shankaran S, Tyson JE, ; Eunice Kennedy Shriver National Institute of Child Health and Human Development Neonatal Research Network. Effect of therapeutic hypothermia initiated after 6 hours of age on death or disability among newborns with hypoxic-ischemic encephalopathy: a randomized clinical trial. JAMA. 2017;318(16):1550-1560. doi:10.1001/jama.2017.14972 29067428 PMC5783566

[97] Makker K, Cortez J, Jha K, . Comparison of extubation success using noninvasive positive pressure ventilation (NIPPV) versus noninvasive neurally adjusted ventilatory assist (NI-NAVA). J Perinatol. 2020;40(8):1202-1210. doi:10.1038/s41372-019-0578-4 31911641 PMC7222927

[98] Marr BL, Mettelman BB, Bode MM, Gross SJ. Randomized trial of 42-day compared with 9-day courses of dexamethasone for the treatment of evolving bronchopulmonary dysplasia in extremely preterm infants. J Pediatr. 2019;211:20-26.e1. doi:10.1016/j.jpeds.2019.04.047 31349916

[99] McMichael ABV, Zimmerman KO, Kumar KR, Ozment CP. Evaluation of effect of scheduled fresh frozen plasma on ECMO circuit life: a randomized pilot trial. Transfusion. 2021;61(1):42-51. doi:10.1111/trf.16164 33269487

[100] Mu TS, Prescott AC, Haischer-Rollo GD, Aden JK, Shapiro JB. Umbilical cord blood use for admission blood tests of VLBW preterm neonates: a randomized control trial. Am J Perinatol. 2023;40(10):1119-1125. doi:10.1542/peds.147.3_MeetingAbstract.1058 34407547

[101] Napolitano N, Dysart K, Soorikian L, Zhang H, Panitch H, Jensen E. Tolerability and efficacy of two doses of aerosolized albuterol in ventilated infants with BPD: a randomized controlled crossover trial. Pediatr Pulmonol. 2021;56(1):97-104. doi:10.1002/ppul.25131 33095509

[102] Nelson MU, Shaw J, Gross SJ. Randomized placebo-controlled trial of topical mupirocin to reduce staphylococcus aureus colonization in infants in the neonatal intensive care unit. J Pediatr. 2021;236:70-77. doi:10.1016/j.jpeds.2021.05.042 34023342

[103] Niebler RA, Chiang-Ching H, Daley K, . Nitric oxide added to the sweep gas of the oxygenator during cardiopulmonary bypass in infants: a pilot randomized controlled trial. Artif Organs. 2021;45(1):22-28. doi:10.1111/aor.13788 32737900

[104] Pandey R, Kanike N, Ibrahim M, . Lactose-free infant formula does not change outcomes of neonatal abstinence syndrome (NAS): a randomized clinical trial. J Perinatol. 2021;41(3):598-605. doi:10.1038/s41372-020-00797-7 32868858

[105] Parker LA, Weaver M, Murgas Torrazza RJ, . Effect of gastric residual evaluation on enteral intake in extremely preterm infants: a randomized clinical trial. JAMA Pediatr. 2019;173(6):534-543. doi:10.1001/jamapediatrics.2019.0800 31034045 PMC6547072

[106] Perretta LJ, Spaight M, Yap V, Perlman J. Randomized study of delayed cord clamping of 30 to 60 seconds in the larger infant born preterm. J Pediatr. 2020;224:153-157. doi:10.1016/j.jpeds.2020.04.058 32651013

[107] Petrikin JE, Cakici JA, Clark MM, . The NSIGHT1-randomized controlled trial: rapid whole-genome sequencing for accelerated etiologic diagnosis in critically ill infants. NPJ Genom Med. 2018;3:6. doi:10.1038/s41525-018-0045-8 29449963 PMC5807510

[108] Phelps DL, Watterberg KL, Nolen TL, ; Eunice Kennedy Shriver National Institute of Child Health and Human Development Neonatal Research Network. Effects of myo-inositol on type 1 retinopathy of prematurity among preterm infants <28 weeks’ gestational age: a randomized clinical trial. JAMA. 2018;320(16):1649-1658. doi:10.1001/jama.2018.14996 30357297 PMC6233812

[109] Pickler RH, Meinzen-Derr J, Moore M, Sealschott S, Tepe K. Effect of Tactile Experience During Preterm Infant Feeding on Clinical Outcomes. Nurs Res. 2020;69(5S)(suppl 1):S21-S28. doi:10.1097/NNR.0000000000000453 32555011 PMC7483367

[110] Pineda R, Smith J, Roussin J, Wallendorf M, Kellner P, Colditz G. Randomized clinical trial investigating the effect of consistent, developmentally-appropriate, and evidence-based multisensory exposures in the NICU. J Perinatol. 2021;41(10):2449-2462. doi:10.1038/s41372-021-01078-7 34012055 PMC8516670

[111] Poola AS, Aguayo P, Fraser JD, . Primary closure versus bedside silo and delayed closure for gastroschisis: a truncated prospective randomized trial. Eur J Pediatr Surg. 2019;29(2):203-208. doi:10.1055/s-0038-1627459 29458229

[112] Pourmoghadam KK, Kubovec S, DeCampli WM, . Passive peritoneal drainage impact on fluid balance and inflammatory mediators: a randomized pilot study. World J Pediatr Congenit Heart Surg. 2020;11(2):150-158. doi:10.1177/2150135119888143 32093557

[113] Ramanathan R, Biniwale M, Sekar K, . Synthetic surfactant CHF5633 Compared with poractant alfa in the Treatment of neonatal respiratory distress syndrome: a multicenter, double-blind, randomized, controlled clinical trial. J Pediatr. 2020;225:90-96.e1. doi:10.1016/j.jpeds.2020.06.024 32553868

[114] Roberts KD, Brown R, Lampland AL, . Laryngeal mask airway for surfactant administration in neonates: a randomized, controlled trial. J Pediatr. 2018;193:40-46.e1. doi:10.1016/j.jpeds.2017.09.068 29174079

[115] Romano-Keeler J, Azcarate-Peril MA, Weitkamp JH, . Oral colostrum priming shortens hospitalization without changing the immunomicrobial milieu. J Perinatol. 2017;37(1):36-41. doi:10.1038/jp.2016.161 27684425 PMC5215726

[116] Rosenfeld WN, Hudak ML, Ruiz N, Gautam S; Jasmine Study Group. Stannsoporfin with phototherapy to treat hyperbilirubinemia in newborn hemolytic disease. J Perinatol. 2022;42(1):110-115. doi:10.1038/s41372-021-01223-2 34635771

[117] Rosterman JL, Pallotto EK, Truog WE, . The impact of neurally adjusted ventilatory assist mode on respiratory severity score and energy expenditure in infants: a randomized crossover trial. J Perinatol. 2018;38(1):59-63. doi:10.1038/jp.2017.154 29072677

[118] Ruoss JL, Bazacliu C, Russell JT, . Routine early antibiotic use in symptomatic preterm neonates: a pilot randomized controlled trial. J Pediatr. 2021;229:294-298.e3. doi:10.1016/j.jpeds.2020.09.056 32979383 PMC8958904

[119] Salas AA, Jerome ML, Chandler-Laney P, Ambalavanan N, Carlo WA. Serial assessment of fat and fat-free mass accretion in very preterm infants: a randomized trial. Pediatr Res. 2020;88(5):733-738. doi:10.1038/s41390-020-1052-x 32634820 PMC7581604

[120] Salas AA, Jerome M, Finck A, Razzaghy J, Chandler-Laney P, Carlo WA. Body composition of extremely preterm infants fed protein-enriched, fortified milk: a randomized trial. Pediatr Res. 2022;91(5):1231-1237. doi:10.1038/s41390-021-01628-x 34183770 PMC8237544

[121] Salas AA, Li P, Parks K, Lal CV, Martin CR, Carlo WA. Early progressive feeding in extremely preterm infants: a randomized trial. Am J Clin Nutr. 2018;107(3):365-370. doi:10.1093/ajcn/nqy012 29529231 PMC6692650

[122] Salas AA, Woodfin T, Phillips V, Peralta-Carcelen M, Carlo WA, Ambalavanan N. Dose-response effects of early vitamin D supplementation on neurodevelopmental and respiratory outcomes of extremely preterm infants at 2 years of age: a randomized trial. Neonatology. 2018;113(3):256-262. doi:10.1159/000484399 29393233 PMC5860938

[123] Schanler RJ, Groh-Wargo SL, Barrett-Reis B, . Improved outcomes in preterm infants fed a nonacidified liquid human milk fortifier: a prospective randomized clinical trial. J Pediatr. 2018;202:31-37.e2. doi:10.1016/j.jpeds.2018.07.005 30195561

[124] Sekar K, Szyld E, McCoy M, . Inhaled nitric oxide as an adjunct to neonatal resuscitation in premature infants: a pilot, double blind, randomized controlled trial. Pediatr Res. 2020;87(3):523-528. doi:10.1038/s41390-019-0643-x 31666688 PMC7223624

[125] Shankaran S, Laptook AR, Pappas A, ; Eunice Kennedy Shriver National Institute of Child Health and Human Development Neonatal Research Network. Effect of depth and duration of cooling on death or disability at age 18 months among neonates with hypoxic-ischemic encephalopathy: a randomized clinical trial. JAMA. 2017;318(1):57-67. doi:10.1001/jama.2017.7218 28672318 PMC5793705

[126] Shankaran S, Bell EF, Laptook AR, ; Eunice Kennedy Shriver National Institute of Child Health, and Human Development Neonatal Research Network. Weaning of moderately preterm infants from the incubator to the crib: a randomized clinical trial. J Pediatr. 2019;204:96-102.e4. doi:10.1016/j.jpeds.2018.08.079 30337189 PMC6326364

[127] Shellhaas RA, Burns JW, Barks JDE, Hassan F, Chervin RD. Maternal voice and infant sleep in the neonatal intensive care unit. Pediatrics. 2019;144(3):e20190288. doi:10.1542/peds.2019-0288 31409691 PMC6855818

[128] Shirk SK, Manolis SA, Lambers DS, Smith KL. Delayed clamping vs milking of umbilical cord in preterm infants: a randomized controlled trial. Am J Obstet Gynecol. 2019;220(5):482.e1-482.e8. doi:10.1016/j.ajog.2019.01.234 30786254

[129] Smith S, Keltner C, Stikes R, Hayes P, Crawford TN. Comparison of axillary and temporal artery thermometry in preterm neonates. J Obstet Gynecol Neonatal Nurs. 2018;47(3):352-361. doi:10.1016/j.jogn.2018.02.013 29625020

[130] Song D, Jegatheesan P, Nafday S, . Patterned frequency-modulated oral stimulation in preterm infants: a multicenter randomized controlled trial. PLoS One. 2019;14(2):e0212675. doi:10.1371/journal.pone.0212675 30817764 PMC6394921

[131] Sood BG, Thomas R, Delaney-Black V, Xin Y, Sharma A, Chen X. Aerosolized Beractant in neonatal respiratory distress syndrome: a randomized fixed-dose parallel-arm phase II trial. Pulm Pharmacol Ther. 2021;66:101986. doi:10.1016/j.pupt.2020.101986 33338661

[132] Soul JS, Bergin AM, Stopp C, ; Boston Bumetanide Trial Group. A pilot randomized, controlled, double-blind trial of bumetanide to treat neonatal seizures. Ann Neurol. 2021;89(2):327-340. doi:10.1002/ana.25959 33201535 PMC8122513

[133] Stokes A, Agthe AG, El Metwally D. Music exposure and maturation of late preterm sleep-wake cycles: a randomised crossover trial. Acta Paediatr. 2018;107(4):582-586. doi:10.1111/apa.14079 28929527

[134] Suterwala MS, Reynolds J, Carroll S, Sturdivant C, Armstrong ES. Using fiberoptic endoscopic evaluation of swallowing to detect laryngeal penetration and aspiration in infants in the neonatal intensive care unit. J Perinatol. 2017;37(4):404-408. doi:10.1038/jp.2016.239 28055025

[135] Travers CP, Carlo WA, Nakhmani A, . Environmental or nasal cannula supplemental oxygen for preterm infants: a randomized cross-over trial. J Pediatr. 2018;200:98-103. doi:10.1016/j.jpeds.2018.03.010 29705116 PMC6109600

[136] Travers CP, Wang T, Salas AA, . Higher- or usual-volume feedings in infants born very preterm: a randomized clinical trial. J Pediatr. 2020;224:66-71.e1. doi:10.1016/j.jpeds.2020.05.033 32464224

[137] Viscardi RM, Terrin ML, Magder LS, . Randomised trial of azithromycin to eradicate *Ureaplasma* in preterm infants. Arch Dis Child Fetal Neonatal Ed. 2020;105(6):615-622. doi:10.1136/archdischild-2019-318122 32170033 PMC7592356

[138] Vittner D, McGrath J, Robinson J, . Increase in oxytocin from skin-to-skin contact enhances development of parent-infant relationship. Biol Res Nurs. 2018;20(1):54-62. doi:10.1177/1099800417735633 29017336

[139] Wallace DK, Kraker RT, Freedman SF, ; Pediatric Eye Disease Investigator Group (PEDIG). Assessment of lower doses of intravitreous bevacizumab for retinopathy of prematurity: a phase 1 dosing study. JAMA Ophthalmol. 2017;135(6):654-656. doi:10.1001/jamaophthalmol.2017.1055 28448664 PMC5521814

[140] Wallace DK, Kraker RT, Freedman SF, ; Pediatric Eye Disease Investigator Group (PEDIG). Short-term outcomes after very low-dose intravitreous bevacizumab for retinopathy of prematurity. JAMA Ophthalmol. 2020;138(6):698-701. doi:10.1001/jamaophthalmol.2020.0334 32324197 PMC7180729

[141] Willeitner A, Anderson M, Lewis J. Highly concentrated preterm formula as an alternative to powdered human milk fortifier: a randomized controlled trial. J Pediatr Gastroenterol Nutr. 2017;65(5):574-578. doi:10.1097/MPG.0000000000001638 29064929

[142] Zuzarte I, Indic P, Barton B, Paydarfar D, Bednarek F, Bloch-Salisbury E. Vibrotactile stimulation: a non-pharmacological intervention for opioid-exposed newborns. PLoS One. 2017;12(4):e0175981. doi:10.1371/journal.pone.0175981 28426726 PMC5398650

[143] Horbar JD, Edwards EM, Greenberg LT, . Racial segregation and inequality in the neonatal intensive care unit for very low–birth-weight and very preterm infants. JAMA Pediatr. 2019;173(5):455-461. doi:10.1001/jamapediatrics.2019.0241 30907924 PMC6503514

[144] Ioannidis JPA, Powe NR, Yancy C. Recalibrating the use of race in medical research. JAMA. 2021;325(7):623-624. doi:10.1001/jama.2021.0003 33492329

[145] McGlothen-Bell K, McGrath JM, Brownell EA, Shlafer RJ, Crawford AD. Applying a Reproductive Justice Lens to Enhance Research Engagement Among Systematically Underrepresented Childbearing Women. Nurs Res. 2023;72(2):132-140. doi:10.1097/NNR.0000000000000639 36508567

[146] Clark LT, Watkins L, Piña IL, . Increasing diversity in clinical trials: overcoming critical barriers. Curr Probl Cardiol. 2019;44(5):148-172. doi:10.1016/j.cpcardiol.2018.11.002 30545650

[147] Hebert PL, Chassin MR, Howell EA. The contribution of geography to black/white differences in the use of low neonatal mortality hospitals in New York City. Med Care. 2011;49(2):200-206. doi:10.1097/MLR.0b013e3182019144 21239954

[148] Brewster RCL, Steinberg JR, Magnani CJ, . Race and ethnicity reporting and representation in pediatric clinical trials. Pediatrics. 2023;151(4):e2022058552. doi:10.1542/peds.2022-058552 36916197

[149] Walker VP, Pavlek LR, Backes CH. Expecting equity: reimagining the delivery of racial/ethnic representation in neonatal clinical trials. J Perinatol. 2022;42(1):1-2. doi:10.1038/s41372-021-01225-0 34615981

[150] Coon ER, Schroeder AR, Lion KC, Ray KN. Disparities by ethnicity in enrollment of a clinical trial. Pediatrics. 2022;149(2):e2021052595. doi:10.1542/peds.2021-052595 35043194

[151] Weiss EM, Guttmann KF, Olszewski AE, . Parental enrollment decision-making for a neonatal clinical trial. J Pediatr. 2021;239:143-149.e3. doi:10.1016/j.jpeds.2021.08.014 34400207 PMC8610170

[152] Weiss EM, Porter KM, Sullivan TR, . Equity concerns across pediatric research recruitment: an analysis of research staff interviews. Acad Pediatr. Published online July 11, 2023. doi:10.1016/j.acap.2023.06.032 37442368 PMC10782814

[153] Kraft SA, Porter KM, Sullivan TR, . Relationship building in pediatric research recruitment: insights from qualitative interviews with research staff. J Clin Transl Sci. 2022;6(1):e138. doi:10.1017/cts.2022.469 36590359 PMC9794958

[154] Rees CA, Stewart AM, Mehta S, . Reporting of participant race and ethnicity in published US pediatric clinical trials from 2011 to 2020. JAMA Pediatr. 2022;176(5):e220142. doi:10.1001/jamapediatrics.2022.0142 35311946 PMC8938892

[155] Srivastav A, Robinson-Ector K, Kipp C, Strompolis M, White K. Who declines to respond to the reactions to race module: findings from the South Carolina Behavioral Risk Factor Surveillance System, 2016-2017. BMC Public Health. 2021;21(1):1703. doi:10.1186/s12889-021-11748-y 34537021 PMC8449882

[156] Eisenhower A, Suyemoto K, Lucchese F, Canenguez K. “Which box should I check?”: examining standard check box approaches to measuring race and ethnicity. Health Serv Res. 2014;49(3):1034-1055. doi:10.1111/1475-6773.12132 24298894 PMC4231584

[157] Flanagin A, Frey T, Christiansen SL, Bauchner H. The reporting of race and ethnicity in medical and science journals: comments invited. JAMA. 2021;325(11):1049-1052. doi:10.1001/jama.2021.2104 33616604

[158] Long JA, Bamba MI, Ling B, Shea JA. Missing race/ethnicity data in Veterans Health Administration based disparities research: a systematic review. J Health Care Poor Underserved. 2006;17(1):128-140. doi:10.1353/hpu.2006.0029 16520522

[159] Mersha TB, Abebe T. Self-reported race/ethnicity in the age of genomic research: its potential impact on understanding health disparities. Hum Genomics. 2015;9(1):1. doi:10.1186/s40246-014-0023-x 25563503 PMC4307746

[160] Chaiyachati BH, Peña MM, Montoya-Williams D. The complicated inadequacy of race and ethnicity data. JAMA Pediatr. 2022;176(7):631-632. doi:10.1001/jamapediatrics.2022.0525 35435954 PMC9359890

[161] US Census Bureau. Decennial census of population and housing questionnaires & instructions. Accessed April 26, 2023, https://www.census.gov/programs-surveys/decennial-census/technical-documentation/questionnaires.2020_Census.html

[162] Flanagin A, Frey T, Christiansen SL; AMA Manual of Style Committee. Updated guidance on the reporting of race and ethnicity in medical and science journals. JAMA. 2021;326(7):621-627. doi:10.1001/jama.2021.13304 34402850

[163] Jones N, Marks R, Ramirez R, Ríos-Vargas M. 2020 Census illuminates racial and ethnic composition of the country. Accessed April 26, 2023, https://www.census.gov/library/stories/2021/08/improved-race-ethnicity-measures-reveal-united-states-population-much-more-multiracial.html

[164] Magaña López M, Bevans M, Wehrlen L, Yang L, Wallen GR. Discrepancies in race and ethnicity documentation: a potential barrier in identifying racial and ethnic disparities. J Racial Ethn Health Disparities. 2016;4(5):812-818. doi:10.1007/s40615-016-0283-3 27631381 PMC5342943

[165] Norris T, Vines PL, Hoeffel EM. The American Indian and Alaska Native population: 2010. *2010 Census Briefs*. January 2012. Accessed November 16, 2023. https://www.census.gov/history/pdf/c2010br-10.pdf

[166] Hoeffel EM, Rastogi S, Kim MO, Shahid H. The Asian population: 2010. *2010 Census Briefs*. March 2012. Accessed November 16, 2023. https://www.census.gov/history/pdf/2010asian-122016.pdf

[167] Moran A, Haines H, Raschke N, . Mind the gap: is it time to invest in embedded researchers in regional, rural and remote health services to address health outcome discrepancies for those living in rural, remote and regional areas? Aust J Prim Health. 2019;25(2):104-107. doi:10.1071/PY18201 30967172

[168] Kowal S, Ng CD, Schuldt R, Sheinson D, Jinnett K, Basu A. Estimating the US baseline distribution of health inequalities across race, ethnicity, and geography for equity-informative cost-effectiveness analysis. Value Health. 2023;26(10):1485-1493. doi:10.1016/j.jval.2023.06.015 37414278

[169] Zahnd WE, Bell N, Larson AE. Geographic, racial/ethnic, and socioeconomic inequities in broadband access. J Rural Health. 2022;38(3):519-526. doi:10.1111/jrh.12635 34792815

