# Supplementary material for: Race and Ethnicity of Infants Enrolled in Neonatal Clinical Trials: A Systematic Review
Source: JAMA Netw Open. 2023 Dec 21;6(12):e2348882. doi: 10.1001/jamanetworkopen.2023.48882 (PMC10739112; doi:10.1001/jamanetworkopen.2023.48882)
Supplement: Supplement 1. — eTable. Characteristics of Included Studies eFigure 1. Flowchart of Included Studies eFigure 2. Race and Ethnicity of Participants eReferences. [file jamanetwopen-e2348882-s001.pdf]

Supplemental Online Content

Lyle AJ, Shaikh H, Oslin E, Gray MM, Weiss EM. Race and ethnicity of infants enrolled in neonatal clinical trials: a systematic review. *JAMA Netw Open*. 2023;6(12):e2348882. doi:10.1001/jamanetworkopen.2023.48882

eTable 1. Characteristics of Included Studies
eFigure 1. Flowchart of Included Studies
eFigure 2. Race and Ethnicity of Participants
eReferences.

This supplemental material has been provided by the authors to give readers additional information about their work.

© 2023 Lyle AJ et al. *JAMA Network Open.*

eTable 1: Characteristics of Included Studies

First author	Year	Study summary	Type of study	Hawker Quality score[20]	Race/ Ethnicity data available
Abbey[23]	2021	RCT comparing immediate ECG use for heart rate assessment versus standard care during delivery room resuscitation for impact on time to heart rate >= 100 bpm among preterm neonates 23-30 weeks GA	Procedural/ interventional	36	No
Alrifal[24]	2017	RCT comparing the use of a parenteral protein calculator clinical decision support system to standard care for attaining appropriate total protein intake in VLBW neonates	Nutrition	34	No
Amaro[25]	2018	RCT evaluating the effect of early caffeine (20 mg/kg followed by 5mg/kg daily) compared to placebo administration on age of first successful extubation in preterm infants 23-30 weeks GA	Drug	34	Yes
Amatya[26]	2017	RCT comparing the rate of successful transition from CPAP to room air among preterm infants (26-32 weeks GA) weaned gradually (every 8 hour decrease by 1cm H2O until 3 cm H2O, then off) versus suddenly	Procedural/ interventional	35	Yes
Anderson-Berry[27]	2017	RCT comparing vitamin D3 dosed at 400 IU/day versus 800 IU/day for effect on serum vitamin D concentration, growth, PTH normalization, and bone density among preterm infants <33 weeks GA	Nutrition	27	Yes

© 2023 Lyle AJ et al. *JAMA Network Open.*

Angeles[28]	2020	Prospective, randomized trial comparing 30% oral dextrose, facilitated tucking, and 30% oral dextrose plus facilitated tucking for effect on pain associated with heel lance among preterm infants born at >=24 weeks GA who required heel lance at 27-36 weeks corrected GA	Mixed	33	No
Angeles[29]	2021	RCT comparing urinary markers of ATP metabolism (hypoxanthine, xanthine, uric acid) among preterm neonates 27 or greater weeks GA who received oral sucrose compared to oral dextrose or standard care prior to painful procedures on day of life 3-7	Drug	36	No
Arnold[30]	2020	A dose-finding randomized trial comparing cycled versus continuous phototherapy for effect on mean peak total serum bilirubin level, total phototherapy hours within 14 days, and predischarge wave V latency BAERs among ELBW infants with hyperbilirubinemia	Procedural/ interventional	35	Yes
Atchley[31]	2019	RCT to investigate the effect of an enhanced compared to standard protein diet on weight gain and fat accretion over the 4-week study period among preterm infants <32 weeks who had attained full enteral feeds	Nutrition	34	No
Backes[32]	2020	Randomized trial comparing rates of CPAP failure between two CPAP modes (Seattle-PAP versus Fisher & Paykel-CPAP) among infants born <30 weeks who were candidates for non-invasive respiratory support	Procedural/ interventional	35	Yes
Balakrishnan[33]	2017	RCT comparing the effects of lower and higher starting dose of parenteral amino acids (1-2 g/kg/day vs. 3-4 g/kg/day) on growth and neurodevelopmental outcomes at 18-24 months corrected age among VLBW infants	Nutrition	35	Yes

© 2023 Lyle AJ et al. *JAMA Network Open.*

Ballengee[34]	2018	RCT comparing the effects of erythromycin versus placebo on esophageal reflux events (measured by pH monitoring) in preterm neonates with clinical signs of gastroesophageal reflux	Drug	31	Yes
Bedwell[35]	2021	RCT evaluating warming human milk continuously versus a hot water bath before feeding among preterm infants <32 weeks for effect on weight gain	Nutrition	34	No
Blakely[36]	2021	RCT evaluating initial laparotomy versus peritoneal drainage for impact on death or neurodevelopmental impairment in preterm infants with necrotizing enterocolitis or isolated intestinal perforation	Procedural/ interventional	36	No
Bogen[37]	2017	RCT assessing the feasibility and effect on weight gain trajectory of providing 24 compared to 20 kilocalorie/ounce formula to infants (>=35 weeks GA) prenatally exposed to methadone	Nutrition	36	Yes
Brandon[38]	2017	RCT comparing initiation of cycled light exposure early (28 weeks PMA) versus late (36 weeks PMA) on weight gain during hospitalization and length of stay among preterm infants (<29 weeks GA)	Procedural/ interventional	33	Yes
Brion[39]	2020	RCT evaluating the impact of advancing human milk fortification based on serial milk nutrient analyses compared to standard advancements (based on weight gain and infant serum nutrient analysis) on weight gain and linear growth from birth to 36 weeks PMA or discharge among infants born at <29 weeks GA or born at <35 weeks GA with birth weight <10th percentile for GA	Nutrition	33	Yes

© 2023 Lyle AJ et al. *JAMA Network Open.*

Brusseau[40]	2020	Randomized trial comparing total morphine treatment days among infants >=35 weeks GA with neonatal abstinence syndrome receiving clonidine versus phenobarbital as adjunctive therapy for inadequate treatment with morphine alone	Drug	32	Yes
Bruzoni[41]	2017	RCT comparing sutureless versus sutured gastroschisis repair on time to extubation and time to full feeds	Procedural/ interventional	30	Yes
Butler-O'Hara[42]	2017	RCT comparing 8-hourly administration of glycerin suppository versus standard care without suppository for effect on phototherapy duration among infants 30-34 weeks requiring this therapy for hyperbilirubinemia	Procedural/ interventional	30	Yes
Calkins[43]	2016	RCT to evaluate the effects of low- versus high-dose (1 versus 3 g/kg/day, respectively) soybean-based intravenous lipid emulsions on development of cholestasis among neonates with gastrointestinal disorders (gastroschisis, omphalocele, small bowel atresia, intestinal perforation, Hirschsprung's disease, volvulus, meconium ileus and/or peritonitis)	Nutrition	31	Yes
Ceyhan-Birsoy[44]	2019	RCT to evaluate the impact of newborn genomic sequencing compared to standard care (state-mandated newborn screening and family history-based genetic counseling) on clinical care, parent and clinician behaviors, and economic outcomes in all healthy and ill newborns (*only NICU infants included in our review*)	Genetics	33	Yes
Choi[45]	2018	Prospective study evaluating the impact of T2 magnetic resonance imaging in predicting postsurgical bleeding for term infants undergoing cardiopulmonary bypass for repair of congenital heart disease	Procedural/ interventional	32	No

© 2023 Lyle AJ et al. *JAMA Network Open.*

Cholette[46]	2016	RCT to assess safety, daily hemoglobin concentration, and oxygen utilization among infants with congenital heart disease requiring surgical management who received red blood cell transfusions according to liberal versus conservative thresholds	Procedural/ interventional	30	No
Chorna[47]	2018	Quasi-randomized prospective study comparing active, suck-contingent exposure to mother's voice to standard care for effect on speech-sound differentiation measured using neurophysiological biomarkers in preterm infants 32-34 weeks GA	Procedural/ interventional	33	Yes
Claassen[48]	2018	RCT comparing rates of extubation failure among preterm infants weighing less than 1250g placed on B&B Bubbler versus BabiPlus CPAP devices following extubation	Procedural/ interventional	28	Yes
Cook[49]	2017	RCT assessing 24% oral sucrose solution compared to placebo for effect on pain (measured using Premature Infant Pain Profile) associated with peripheral IV insertion among preterm neonates at least 27 weeks GA	Drug	36	Yes
Cooper[50]	2020	RCT comparing 15-20 minutes of daily developmentally appropriate motor activities to standard care among preterm infants born at <29 weeks GA and with current PMA of >34 weeks on full feeds and nearing discharge for effect on body composition at one year of life	Procedural/ interventional	30	No
Corrigan[51]	2020	Randomized trial comparing exposure of preterm infants to music therapy (recorded maternal singing or heartbeat) to standard care for effect on pain measured via premature infant pain profile (PIPP) during and after retinopathy of prematurity exam	Procedural/ interventional	34	Yes

© 2023 Lyle AJ et al. *JAMA Network Open.*

Cummings[52]	2020	RCT comparing aerosolized calfactant administration to usual care for effect on frequency of clinically required intubation and surfactant instillation among infants with respiratory distress syndrome requiring non-invasive respiratory support	Drug	32	No
Czarnecki[53]	2019	RCT comparing parent/nurse controlled analgesia versus continuous opioid infusion on the outcome of average opioid consumption for pain control in post-operative infants	Procedural/ interventional	35	Yes
Czynski[54]	2020	Randomized trial comparing treatment of neonatal abstinence syndrome with morphine versus methadone for impact on neurodevelopmental outcomes, measured as NICU Network Neurobehavioral Scale at hospital discharge and Bayley Scale and Child Behavior Checklist at 18 months	Drug	31	No
Daniel[55]	2020	RCT comparing aromatherapy plus standard care versus standard care alone for effect on duration of therapy and length of stay among neonates >=36 weeks GA with opioid withdrawal syndrome	Procedural/ interventional	31	No
Davidson[56]	2019	RCT to evaluate the impact of olfactory stimulation with mother's milk compared to water (sham) on post-menstrual age at attainment of full oral feeds among preterm infants born 28-33 weeks GA	Nutrition	32	No
Davis[57]	2018	RCT evaluating safety and efficacy of methadone compared to morphine for length of hospital stay among term infants with neonatal abstinence syndrome	Drug study	35	Yes

© 2023 Lyle AJ et al. *JAMA Network Open.*

Detmer[58]	2020	RCT comparing music therapy (multimodal neurological enhancement) to standard care for effect on neurodevelopment measured using the Mullen Scales of Early Learning 3 months post-discharge among preterm infants born at 31 weeks GA or less	Procedural/ interventional	33	Yes
DuPont[59]	2021	RCT assessing the safety, feasibility, and neurodevelopmental impact (measured via Bayley Scale at 8-12 months corrected age) of administering one dose of darbepoetin alpha compared to placebo to infants at least 34 weeks GA with mild neonatal encephalopathy	Drug study	36	No
Elzein[60]	2020	RCT comparing nitric oxide versus placebo added to oxygenator during cardiopulmonary bypass during Norwood procedure for effect on serum markers of ischemia/reperfusion injury	Drug study	32	No
Emery[61]	2018	RCT evaluating the differential impact of 2 music therapy regimens (intensive-intermittent, standard spaced) on developmental milestone acquisition over the 4 week study period in infants >44 weeks PMA	Procedural/ interventional	33	Yes
Eze[62]	2017	RCT comparing a sprinting (gradually increasing spontaneous breathing time) versus non-sprinting (weaning pressure down) protocol for impact on successful weaning from CPAP to room air among preterm infants (<31 weeks GA)	Procedural/ interventional	34	Yes
Foglia[63]	2019	Safety and feasibility trial of intubation prior to umbilical cord clamping at delivery in infants >= 36 weeks GA with congenital diaphragmatic hernia	Procedural/ interventional	33	No

© 2023 Lyle AJ et al. *JAMA Network Open.*

Forde[64]	2020	RCT comparing urinary biomarkers of energy utilization and oxidative stress among preterm infants born at 24-36 weeks GA who received 1 hour of kangaroo mother care versus incubator care on days 3 and 4 of life	Procedural/ interventional	35	Yes
Frost[65]	2021	RCT to assess feasibility of providing concentrated emulsified long-chain polyunsaturated fatty acids (LCPUFA) in two different doses compared to placebo and to assess the impact on blood LCPUFA concentrations among VLBW preterm infants	Nutrition	36	No
Gautam[66]	2020	RCT comparing the effect of platelet transfusion during the rewarming phase of cardiopulmonary bypass to transfusion at the end of bypass on need for postoperative blood product resuscitation among neonates undergoing elective open heart surgery	Procedural/ interventional	30	No
Gaynor[67]	2018	RCT evaluating the effect of remote ischemic preconditioning (brief periods of ischemia to tissues resistant to ischemia, like skeletal muscle) compared to sham procedure on postoperative white matter injury among term neonates undergoing cardiopulmonary bypass	Procedural/ interventional	31	No
Gerges[68]	2018	RCT comparing initiation of oral feeds at 30 versus 33 weeks PMA for effect on time to full oral feedings or hospital discharge for preterm infants born at <29 weeks GA	Nutrition	33	Yes
Glass[69]	2017	RCT comparing oropharyngeal administration of colostrum versus sterile water on salivary IgA levels and incidence of late-onset sepsis and necrotizing enterocolitis among VLBW neonates	Nutrition	28	No

© 2023 Lyle AJ et al. *JAMA Network Open.*

Graham[70]	2019	RCT to evaluate the impact of intraoperative methylprednisone compared to placebo on postoperative morbidity and mortality among term infants undergoing cardiopulmonary bypass for repair of congenital heart disease	Drug study	36	Yes
Gray[71]	2016	RCT comparing every three-hour to every six-hour oral feeding attempts for effect on duration to full oral feeds among neonates born preterm (<=33 weeks GA) showing feeding cues	Nutrition	34	No
Gupta[72]	2020	RCT comparing two concentrations of soy-based intravenous lipid emulsions for impact on rate of rise of direct bilirubin and incidence of intestinal failure-associated fatty liver disease among infants with gastrointestinal surgical disorders	Nutrition	36	Yes
Hammer[73]	2019	RCT to evaluate the efficacy of intravenous acetaminophen at two different dosing regimens compared to placebo in controlling pain among neonates >= 28 weeks and infants postoperatively or following trauma	Drug study	36	Yes
Hammond[74]	2021	RCT evaluating the amino acid composition of total parenteral nutrition (18% vs. 12.5% amino acids (experimental vs standard, respectively)) among VLBW infants for impact on sleep state distribution after achieving full feeds	Nutrition	35	Yes
Hart[75]	2017	RCT evaluating Velcro compared to twill tie (standard of care) for securing tracheostomy tubes for effect on skin-related complications and accidental decannulation among patients <=21 years (57 total patients; 40 who received a neonatal tracheostomy tube)	Procedural/ interventional	35	Yes

© 2023 Lyle AJ et al. *JAMA Network Open.*

Havranek[76]	2019	RCT to evaluate the impact of prone compared to supine positioning on need for respiratory intervention following scheduled cesarean delivery of vigorous newborns at 37-42 weeks	Procedural/ interventional	34	No
Hibbs[77]	2018	RCT evaluating the effectiveness of two different vitamin D dosing strategies (400 IU/day cholecalciferol until 6 months corrected age versus diet-limited supplementation) for preventing recurrent wheezing within 12 months corrected age among Black preterm infants (born 28-36 weeks GA)	Drug	33	Yes
Jadcherla[78]	2020	RCT comparing four weeks of a feeding bundle (total fluid limitation, feeds over 30 minutes in right lateral position, supine postprandial position) plus acid suppression versus acid suppression alone for impact on GERD symptom scores and independent oral feeding skills among infants 34-60 weeks post-menstrual age	Nutrition	36	Yes
Jakubowitz[79]	2018	Trial evaluating the accuracy and precision of transcutaneous CO_2 and O_2 monitoring electrodes set to various temperatures lower than the standard 42ºC	Procedural/ interventional	30	No
Jooste[80]	2018	RCT to evaluate the impact of intraoperative antithrombin administration compared to placebo on postoperative coagulation status among infants birth to 7 months undergoing cardiopulmonary bypass for repair of congenital heart disease	Procedural/ interventional	31	No
Josephson[81]	2020	RCT comparing umbilical cord milking versus immediate cord clamping among extremely preterm infants for effect on initial hemoglobin level and need for blood transfusions in the first 28 days of life	Procedural/ interventional	34	Yes

© 2023 Lyle AJ et al. *JAMA Network Open.*

Juul[82]	2020	RCT comparing administration of intravenous high-dose erythropoietin versus placebo in extremely preterm infants for impact on composite outcome of death or severe neurodevelopmental impairment at 22 to 26 months of age	Drug	34	Yes
Kakkilaya[83]	2020	Randomized trial comparing immediate versus stepwise discontinuation of CPAP for effect on total CPAP days among preterm infants born at 32 or fewer gestational weeks	Procedural/ interventional	36	Yes
Katheria[84]	2021	RCT comparing ECG integrated into Panda Bed Warmer versus stand-alone cardiac monitor for time to heart rate display following electrode placement during resuscitation of preterm infants born at 23-32 weeks GA	Procedural/ interventional	29	No
Katheria[85]	2017	Evaluation of a subset of infants included in the Nuero-monitoring of Preterm Newborn Brain During Birth Resuscitation (Neu-Prem) RCT to assess whether displaying compared to not displaying ECG information during delivery room resuscitation impacted time to first change in FiO2, first positive pressure ventilation, first increase in airway pressure, and first intubation among preterm infants 23-32 weeks GA	Procedural/ interventional	33	No
Kim[86]	2020	RCT evaluating the effect of withholding versus not withholding the initial 48 hours of empiric antibiotics in low-risk preterm infants born at 28-34 weeks GA on microbiome diversity measured through DNA sequencing of fecal sampling	Drug	29	No

© 2023 Lyle AJ et al. *JAMA Network Open.*

Kingsmore[87]	2019	RCT to evaluate the diagnostic effectiveness and clinical utility of two rapid genomic sequencing methods (rapid whole genome sequencing, rapid whole exome sequencing) among infants <4 months old admitted to the NICU, PICU, or CVICU with features suggestive of a genetic illness	Genetics	33	Yes
Kirpalani[88]	2020	RCT comparing higher or lower threshold for red-cell transfusion (each threshold based on corrected GA and respiratory support) among extremely low birth weight neonates for impact on composite outcome of death or neurodevelopmental impairment at 22 to 26 months of age	Procedural/ interventional	35	Yes
Kochan[89]	2018	RCT to evaluate the impact of elevated compared to flat head positioning on incidence of intraventricular hemorrhage among ELBW neonates	Procedural/ interventional	33	No
Kotloff[90]	2018	RCT to evaluate the impact of mupirocin compared to no treatment on *Staphylococcus aureus* decolonization among NICU infants <24 months old	Drug	36	Yes
Kraft[91]	2017	RCT comparing sublingual buprenorphine versus oral morphine for effect on treatment duration for term neonates with neonatal opioid withdrawal syndrome	Drug	32	Yes
Kumar[92]	2017	Prospective trial comparing Enfamil versus Similac human milk fortifier for effect on weight gain and incidence of metabolic acidosis among preterm neonates born <=31 weeks who were exclusively fed human milk	Nutrition	28	Yes

© 2023 Lyle AJ et al. *JAMA Network Open.*

Lafferty[93]	2021	RCT evaluating exposure to Mozart for two weeks compared to no exposure on outcome of time to regain birthweight among preterm infants born at 28-31 weeks GA	Procedural/ interventional	34	No
Lam[94]	2019	RCT evaluating CPAP extended for 2 additional weeks versus standard discontinuation of CPAP for impact on functional residual capacity at the end of the 2-week treatment period and at hospital discharge among neonates born at 32 or fewer weeks GA requiring CPAP for at least 24 hours	Procedural/ interventional	35	Yes
Lambert[95]	2017	Prospective trial evaluating the safety and feasibility of a passive range of motion exercise program for infants who had undergone a stage 1 palliation procedure for single ventricle physiology	Procedural/ interventional	32	Yes
Laptook[96]	2017	RCT assessing survival and neurodevelopmental outcomes at 18-22 months among infants (>=36 weeks GA) with hypoxic ischemic encephalopathy who received therapeutic hypothermia initiated at 6 to 24 hours after birth compared to standard care (no hypothermia initiated after 6 hours of life)	Procedural/ interventional	30	Yes
Makker[97]	2020	RCT comparing rates of initial extubation success in VLBW preterm infants born between 24-32 weeks extubated in the first 14 days of life and transitioned to NIPPV versus NI-NAVA	Procedural/ interventional	35	Yes
Marr[98]	2019	RCT to evaluate the impact of a 42-day compared to a 9-day course of dexamethasone given between 10-21 days of life on intact survival at 7 years of life (normal neurologic exam, IQ >70, attending school without supplemental educational support) among infants born at 24-27 weeks GA meeting respiratory criteria for evolving BPD	Drug	34	Yes

© 2023 Lyle AJ et al. *JAMA Network Open.*

McMichael[99]	2020	Pilot RCT evaluating transfusion of fresh frozen plasma every 48 hours compared to usual care for frequency of ECMO circuit change among patients 18 years old or younger, including 22 infants <=6 months	Procedural/ interventional	33	No
Mu[100]	2021	RCT evaluating the effect of drawing admission labs from umbilical cord versus the infant on hemoglobin concentration at 24 hours of life among VLBW infants	Procedural/ interventional	35	Yes
Napolitano[101]	2020	RCT evaluating the tolerability and efficacy of two different doses of albuterol (1.25 and 2.5 mg) for improving expiratory flow (pre- to immediately post-treatment) among ventilated preterm infants born at <32 weeks GA and diagnosed with severe bronchopulmonary dysplasia at 36 weeks corrected GA	Drug	32	No
Nelson[102]	2021	RCT evaluating efficacy of topical mupirocin compared to placebo for reducing *Staphylococcus aureus* colonization (measured via a negative PCR screen 2 weeks after treatment) among all NICU infants	Drug	33	No
Niebler[103]	2020	RCT comparing change in platelet counts and activation over the course of cardiopulmonary bypass during surgery among infants who received nitric oxide versus placebo added to the oxygenator sweep gas	Drug	29	No
Pandey[104]	2020	RCT evaluating lactose-free to lactose-containing formula for impact on cumulative morphine dose required among infants >=36 weeks GA receiving treatment for neonatal abstinence syndrome	Nutrition	35	Yes

© 2023 Lyle AJ et al. *JAMA Network Open.*

Parker[105]	2019	RCT to evaluate the impact of omitting compared to performing gastric residual volume measurements on weekly enteral nutrition volume over the 6-week study period among infants born at <= 32 weeks GA with a birth weight <1250g and receiving feeds within 72 hours after birth	Nutrition	35	Yes
Parretta[106]	2020	RCT comparing delayed cord clamping for 30 versus 60 seconds in preterm infants born at 28-34 weeks GA for impact on hematocrit measurement on admission to the NICU	Procedural/ interventional	34	No
Petrikin[107]	2018	RCT comparing differential rates of attaining a genetic diagnosis within 28 days from testing between infants receiving rapid whole genome sequencing plus standard genetic tests compared to standard genetic tests alone	Genetics	33	Yes
Phelps[108]	2018	RCT comparing the safety and efficacy of *myo*-inositol to placebo for reducing incidence of ROP requiring surgical intervention among preterm infants born <28 weeks GA	Drug	34	Yes
Pickler[109]	2020	RCT comparing touch or holding during feeding to standard feeding care among preterm infants born at 32 or fewer weeks GA for impact on time to full oral feeding	Nutrition	35	Yes
Pineda[110]	2021	RCT comparing exposure to age-appropriate sensory stimulation versus standard care for impact on neurodevelopment measured as Ages and Stages Questionnaire score at 1 year corrected age among preterm infants born at or before 32 weeks GA	Procedural/ interventional	36	Yes
Poola[111]	2018	RCT to evaluate the impact of primary closure compared to bedside silo and delayed closure on length of hospital stay among infants born at >= 34 weeks GA with gastroschisis	Procedural/ interventional	33	No

© 2023 Lyle AJ et al. *JAMA Network Open.*

Pourmoghadam[112]	2020	RCT comparing placement of a prophylactic passive peritoneal drain to no placement among infants requiring cardiopulmonary bypass for surgical correction of congenital heart disease for impact on time to negative total fluid balance	Procedural/ interventional	36	No
Ramanathan[113]	2020	RCT evaluating CHF5633 synthetic surfactant versus proactant alfa for impact on oxygen requirement, respiratory severity scores, development of BPD, and death at 36 weeks PMA in preterm newborns born at 24-29 weeks GA with respiratory distress syndrome	Drug	36	No
Roberts[114]	2017	RCT comparing the effects of surfactant delivery via a laryngeal mask airway followed by CPAP to CPAP alone on the need for intubation and mechanical ventilation in the first 7 days of life among preterm infants 28-35 weeks GA	Procedural/ interventional	33	No
Romano-Keeler[115]	2016	RCT comparing the effect of providing 0.1 ml oral colostrum beginning within 48 hours of birth to not providing colostrum on salivary immune peptide sequence representation and oral microbiome diversity among preterm infants <32 weeks GA	Procedural/ interventional	28	Yes
Rosenfeld[116]	2021	RCT evaluating safety and efficacy of tin mesoporphyrin compared to placebo for reducing total serum bilirubin among infants born at least 35 weeks GA receiving phototherapy in the first 48 hours of life for hyperbilirubinemia related to hemolytic disease	Mixed	33	Yes

© 2023 Lyle AJ et al. *JAMA Network Open.*

Rosterman[117]	2017	Randomized crossover trial assessing the effect of neurally adjusted ventilatory assist compared to synchronized intermittent mandatory ventilation on respiratory severity scores among all infants >22 weeks GA	Procedural/ interventional	30	No
Ruoss[118]	2020	Pilot RCT evaluating withholding vs administering antibiotics immediately after birth for preterm infants born <33 weeks GA for effect on composite outcome of late onset sepsis, bronchopulmonary dysplasia, necrotizing enterocolitis, and death	Drug	29	No
Salas[119]	2020	RCT evaluating percent body fat at 3 months corrected age among preterm infants born 28-32 weeks GA who had versus did not have body composition reports made available to their treating physicians	Nutrition	35	Yes
Salas, Jerome, Finck, et al.[120]	2021	RCT comparing supplemental protein included in fortified human milk feeds versus a standard level of protein for effect on percent body fat at 30-32 weeks PMA and 3 months corrected age among preterm infants born at 25-28 weeks GA	Nutrition	33	Yes
Salas, Li, Parks, et al.[121]	2018	RCT evaluating the feasibility and efficacy of early (starting on day of life 1) compared to delayed (starting on day of life 5) feeding for effect on number of full enteral feeding days in the first month of life among preterm infants <=28 weeks GA	Nutrition	30	Yes
Salas, Woodfin, Phillips, et al.[122]	2018	RCT comparing two vitamin D dose schemes (200 or 800 IU/day) and placebo for effect on Bayley III cognitive scores at 22-26 months of age among preterm infants born at 23-27 weeks GA	Drug	31	Yes

© 2023 Lyle AJ et al. *JAMA Network Open.*

Schanler[123]	2018	RCT comparing the effects of acidified verses non-acidified liquid human milk fortifier on weight gain among preterm infants born <=32 weeks GA	Nutrition	35	Yes
Sekar[124]	2019	RCT to evaluate the impact of delivery room resuscitation with inhaled nitric oxide compared to placebo on total exposure to supplemental oxygen as well as heart rate, oxygen saturation, and need for intubation in the first 20 minutes of life among infants 25-31 weeks GA requiring positive pressure ventilation with supplemental oxygen at birth	Procedural/ interventional	34	Yes
Shankaran[125]	2017	RCT comparing the effects of four combinations of therapeutic cooling depths and durations (2x2 factorial design to 33.5C or 32.0C and for 72 or 120 hours) on death and neurodevelopmental outcomes at 18-22 months among neonates (>=36 weeks GA) with hypoxic ischemic encephalopathy	Procedural/ interventional	36	Yes
Shankaran[126]	2018	RCT comparing the effect of weaning from the incubator at 1600g verses 1800g on length of hospital stay among preterm infants born at 29-33 weeks GA	Procedural/ interventional	34	Yes
Shellaas[127]	2019	Prospective trial to evaluate the impact of exposure to mother's voice (recorded book reading) compared to standard NICU ambient noise on sleep structure and the probability of awakening among infants born at 33-41 weeks GA	Procedural/ interventional study	33	No
Shirk[128]	2019	RCT to evaluate the impact of umbilical cord milking compared to delayed cord clamping on initial hematocrit concentration among infants born at 23 to 34 weeks GA	Procedural/ interventional	36	Yes

© 2023 Lyle AJ et al. *JAMA Network Open.*

Smith[129]	2018	Trial evaluating non-inferiority of infrared temporal artery thermometer readings compared to axillary temperature readings in a cohort of preterm neonates between 28 to 36 weeks PMA	Procedural/ interventional	35	Yes
Song[130]	2019	RCT to evaluate the impact of a pulsatile pacifier (NTrainer system) compared to standard, non-pulsatile pacifier on time to full oral feeds among infants born at 26-30 weeks GA	Nutrition	36	Yes
Sood[131]	2020	Randomized trial evaluating feasibility, safety, and efficacy for reducing need for intubation of four dosing schedules of aerosolized surfactant using two nebulizer devices in preterm infants born at 24-36 weeks GA with respiratory distress syndrome in the first 24 hours of life	Procedural/ interventional	35	Yes
Soul[132]	2020	RCT of bumetanide added to phenobarbital vs. phenobarbital alone for treating neonatal seizures among infants 34-44 weeks corrected GA	Drug	35	Yes
Stokes[133]	2017	Randomized crossover trial evaluating the effect of exposure to music on sleep-wake cycles (measured using continuous amplitude-integrated electroencephalography) in preterm neonates born 32-36 weeks GA	Procedural/ interventional	33	Yes
Suterwala[134]	2017	RCT evaluating the safety of FEES and reliability of FEES compared to videofluoroscopic swallowing study for identifying laryngeal penetration and tracheal aspiration in infants at least 37 weeks post-menstrual age	Procedural/ interventional	33	Yes
Travers[135]	2018	Trial comparing the effect of environmental oxygen delivery via servo-controlled incubator versus nasal cannula on episodes of intermittent hypoxemia among preterm neonates <37 weeks GA	Procedural/ interventional	35	Yes

© 2023 Lyle AJ et al. *JAMA Network Open.*

Travers[136]	2020	RCT comparing higher volume (180-220 ml/kg/d) to usual-volume (140-160 ml/kg/d) feeds for impact on growth velocity from study randomization until 36 weeks PMA or hospital discharge among infants with birth weight 1001-2500g	Nutrition	36	Yes
Viscardi[137]	2020	RCT evaluating intravenous azithromycin compared to placebo for outcome of survival to discharge with 3 negative *Ureaplasma* tracheal aspirate cultures among preterm infants born at 24-28 weeks GA	Procedural/ interventional	35	Yes
Vittner[138]	2017	Prospective trial evaluating the impact of skin-to-skin care on infant and parent salivary oxytocin and cortisol levels and parental anxiety levels before, during, and after the intervention as well as observed parent-infant synchrony and responsiveness after the intervention among stable preterm infants born at 30-34 weeks GA	Procedural/ interventional	36	Yes
Wallace[139]	2017	Dose de-escalation trial in which lower doses of bevacizumab were sequentially administered to infants with retinopathy of prematurity in order to evaluate whether doses lower than in standard use may provide effective treatment	Drug	29	No
Wallace[140]	2020	Dose-escalation study with objective of determining the lowest effective dose of intravitreous bevacizumab for treating retinopathy of prematurity	Drug	26	No
Willeitner[141]	2017	RCT comparing the effects of the concentrated preterm formula Similac Special Care 30 with iron to powdered Similac Human Milk Fortifier on weight gain among VLBW neonates	Nutrition	35	Yes

© 2023 Lyle AJ et al. *JAMA Network Open.*

Zuzarte[142]	2017	Prospective trial evaluating the impact of stochastic vibrotactile stimulation on hyperirritability among neonates receiving pharmacologic treatment for neonatal opioid withdrawal syndrome	Procedural/ interventional	29	No

BPD = bronchopulmonary dysplasia
ECMO = extracorporeal membrane oxygenation
ELBW = extremely low birth weight (<1000g)
FEES = fiberoptic endoscopic evaluation of swallowing
GA = gestational age
NICU = neonatal intensive care unit
NI-NAVA = non-invasive neurally adjusted ventilatory assist
NIPPV = non-invasive positive pressure ventilation
PMA = post-menstrual age
RCT = randomized controlled trial
VLBW = very low birth weight (<1500g)

All studies were entered into Cochrane CENTRAL 2017-2021. There was a range of availability via electronic publication (2016-2021) and final publication year (2017-2023). Year documented in this table is based on first availability via electronic publication.

Mixed studies are those comparaing pharmacotherapy to procedural interventions.
Nutrition includes feeding studies.

Hawker score was determined the sum of 9 components each scored from 1 (very poor) to 4 (good) resulting in a total score between 9 and 36. Scores were considered high (31-36), moderate (25-30), low (20-24), or very low (9-19).[21,22]

© 2023 Lyle AJ et al. *JAMA Network Open.*

eFigure 1: PRISMA Flowchart of Included Studies

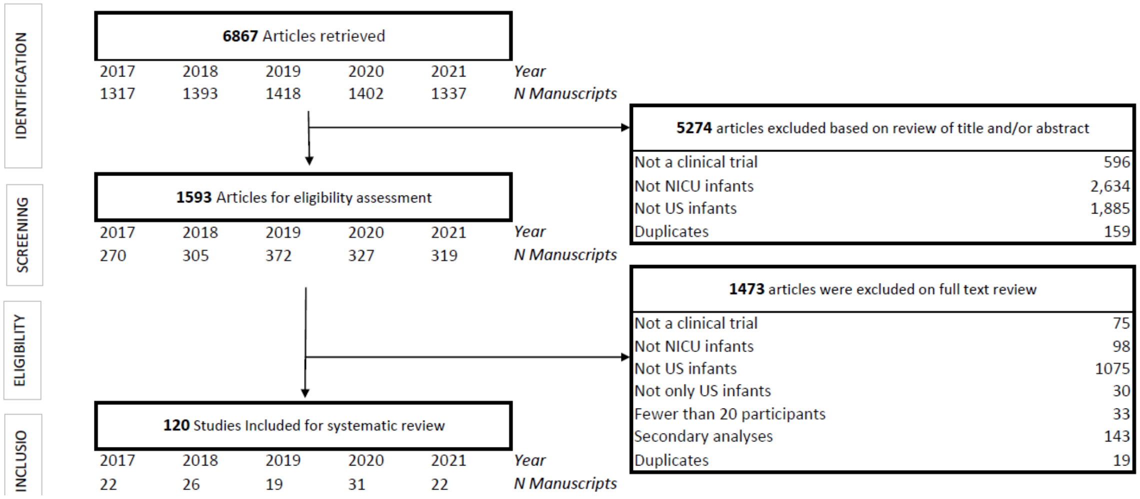

© 2023 Lyle AJ et al. *JAMA Network Open.*

eFigure 2: Race and Ethnicity of Participants

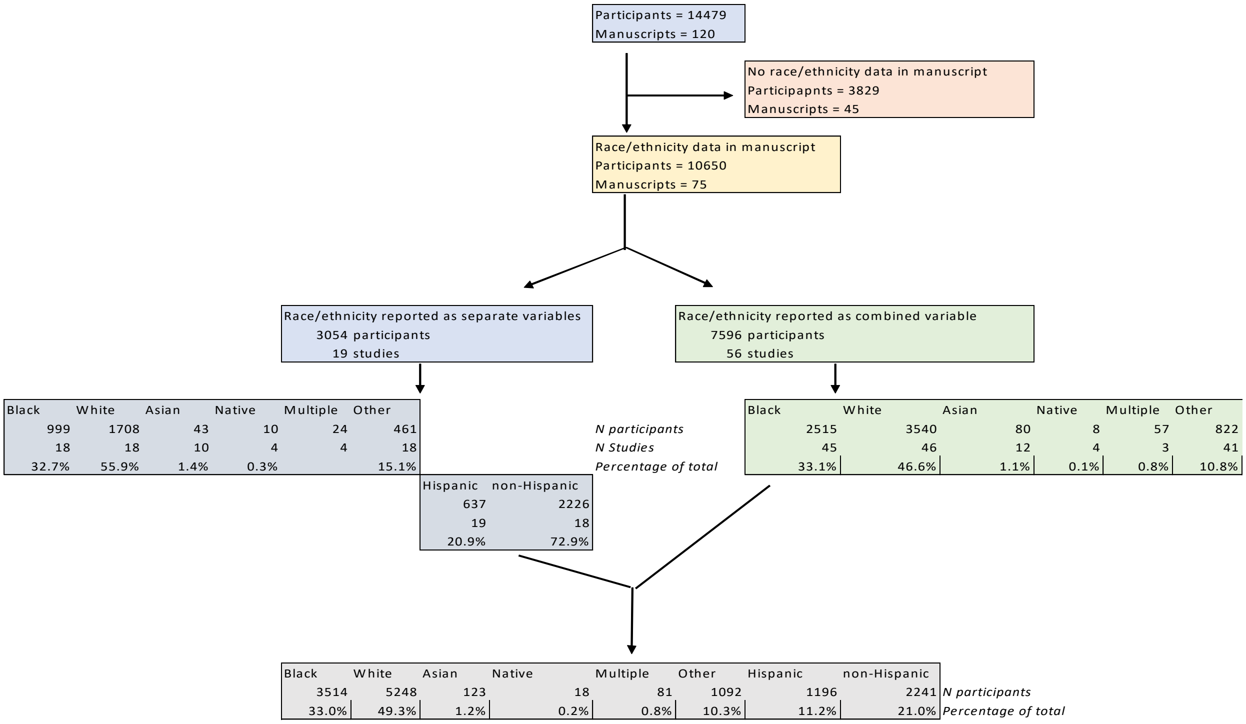

Counts are total numbers of N particpants in N manuscripts reported in each race and Ethnicity category.

1-variable race and Ethnicity categories sum is 2 greater than total N due to Table inconsistencies from Ceyhan-Birsoy[44]
2-variable race categories sum is 5 fewer than total N due to Table inconsistencies from Marr[98]

Note that for manuscripts reporting race and Ethnicity as separate variables (left half), it was typically impossible to align the two. For example, the reporting separately of race and Ethnicity did not allow to reader to determine numbers of “white Hispanic” vs. “Other Hispanic” vs. “multiple races Hispanic” in the study population.

It was typically not possible to determine whether the categorization of “Other” was self-selected by parents or was assigned by research team; included in “Other” category were the specific labels: Missing, Non-White, Not Reported, Not Specified, Other, Other/Unknown, Presumed Non-White, Unknown, Unknown Ethnicity, Unknown, Not Reported, Unknown/Undetermed Ethnicity, and Unreported. Also included in “Other” were those for which our team presumed this determination: for example if a manuscript claimed N=100 participants and stated that N=80 were "white" then we included N=20 in this category.

© 2023 Lyle AJ et al. *JAMA Network Open.*

eReferences

1. Paskett ED, Reeves KW, McLaughlin JM, et al. Recruitment of minority and underserved populations in the United States: the Centers for Population Health and Health Disparities experience. Contemp Clin Trials. 2008;29(6):847-861. Medline:18721901 doi:10.1016/j.cct.2008.07.006
2. Natale JE, Lebet R, Joseph JG, et al; Randomized Evaluation of Sedation Titration for Respiratory Failure (RESTORE) Study Investigators. Racial and ethnic disparities in parental refusal of consent in a large, multisite pediatric critical care clinical trial. J Pediatr. 2017;184:204-208.e1. Medline:28410087 doi:10.1016/j.jpeds.2017.02.006
3. Paquette E, Shukla A, Duyar S. Social Determinants of Research Engagement and Implications for Precision Medicine: Sociodemographic Factors Associated with Differential Enrollment in a Pediatric Critical Care Biorepository. Pediatric Academic Societies; 2018.
4. Liu L, Krailo M, Reaman GH, Bernstein L; Surveillance, Epidemiology and End Results Childhood Cancer Linkage Group. Childhood cancer patients' access to cooperative group cancer programs: a population-based study. Cancer. 2003;97(5):1339-1345. Medline:12599243 doi:10.1002/cncr.11192
5. Foglia EE, Nolen TL, DeMauro SB, et al; Eunice Kennedy Shriver National Institute of Child Health and Human Development Neonatal Research Network. Short-term outcomes of infants enrolled in randomized clinical trials vs those eligible but not enrolled. JAMA. 2015;313(23):2377-2379. Medline:26080344 doi:10.1001/jama.2015.5734
6. Weiss EM, Olszewski AE, Guttmann KF, et al. Parental factors associated with the decision to participate in a neonatal clinical trial. JAMA Netw Open. 2021;4(1):e2032106. Medline:33433595 doi:10.1001/jamanetworkopen.2020.32106
7. Murphy SL, Xu J, Kochanek KD, Curtin SC, Arias E. Deaths: final data for 2015. Natl Vital Stat Rep. 2017;66(6):1-75. Medline:29235985
8. Glazer KB, Zeitlin J, Egorova NN, et al. Hospital quality of care and racial and ethnic disparities in unexpected newborn complications. Pediatrics. 2021;148(3):e2020024091. Medline:34429339 doi:10.1542/peds.2020-024091
9. Anderson JG, Rogers EE, Baer RJ, et al. Racial and ethnic disparities in preterm infant mortality and severe morbidity: a population-based study. Neonatology. 2018;113(1):44-54. Medline:29073624 doi:10.1159/000480536
10. Wallace ME, Mendola P, Kim SS, et al. Racial/ethnic differences in preterm perinatal outcomes. Am J Obstet Gynecol. 2017;216(3):306.e1-306.e12. Medline:27865977 doi:10.1016/j.ajog.2016.11.1026
11. Karvonen KL, Baer RJ, Rogers EE, et al. Racial and ethnic disparities in outcomes through 1 year of life in infants born prematurely: a population based study in California. J Perinatol. 2021;41(2):220-231. Medline:33514879 doi:10.1038/s41372-021-00919-9
12. Beck AF, Edwards EM, Horbar JD, Howell EA, McCormick MC, Pursley DM. The color of health: how racism, segregation, and inequality affect the health and well-being of preterm infants and their families. Pediatr Res. 2020;87(2):227-234. Medline:31357209 doi:10.1038/s41390-019-0513-6
13. Janevic T, Zeitlin J, Auger N, et al. Association of race/ethnicity with very preterm neonatal morbidities. JAMA Pediatr. 2018;172(11):1061-1069. Medline:30208467 doi:10.1001/jamapediatrics.2018.2029

© 2023 Lyle AJ et al. *JAMA Network Open.*

14. American Academy of Pediatrics Board of Directors and Executive Committee. AAP perspective: race-based medicine. Pediatrics. 2021;148(4):e2021053829. Medline:34353893 doi:10.1542/peds.2021-053829
15. Lett E, Asabor E, Beltrán S, Cannon AM, Arah OA. Conceptualizing, contextualizing, and operationalizing race in quantitative health sciences research. Ann Fam Med. 2022;20(2):157-163. Medline:35045967 doi:10.1370/afm.2792
16. National Institutes of Health. NIH's definition of a clinical trial. Accessed November 2, 2021. https://grants.nih.gov/policy/clinical-trials/definition.htm
17. Weiss EM. Enrollment rates and demographic characteristics of research participants in US NICUs: a systematic review. Accessed November 2, 2021, https://www.crd.york.ac.uk/prospero/display_record.php?RecordID=240234
18. Centers for Disease Control and Prevention. National Vital Statistics System: birth data. Accessed June 29, 2023. https://www.cdc.gov/nchs/nvss/births.htm
19. Harris PA, Taylor R, Minor BL, et al; REDCap Consortium. The REDCap consortium: building an international community of software platform partners. J Biomed Inform. 2019;95:103208. Medline:31078660 doi:10.1016/j.jbi.2019.103208
20. Hawker S, Payne S, Kerr C, Hardey M, Powell J. Appraising the evidence: reviewing disparate data systematically. Qual Health Res. 2002;12(9):1284-1299. Medline:12448672 doi:10.1177/1049732302238251
21. Cavolo A, Dierckx de Casterlé B, Naulaers G, Gastmans C. Physicians' attitudes on resuscitation of extremely premature infants: a systematic review. Pediatrics. 2019;143(6):e20183972. Medline:31076541 doi:10.1542/peds.2018-3972
22. Nathe JM, Oskoui TT, Weiss EM. Parental views of facilitators and barriers to research participation: systematic review. Pediatrics. 2023;151(1):e2022058067. Medline:36477217 doi:10.1542/peds.2022-058067
23. Abbey NV, Mashruwala V, Weydig HM, et al. Electrocardiogram for heart rate evaluation during preterm resuscitation at birth: a randomized trial. Pediatr Res. 2022;91(6):1445-1451. Medline:34645954 doi:10.1038/s41390-021-01731-z
24. Alrifai MW, Mulherin DP, Weinberg ST, Wang L, Lehmann CU. Parenteral protein decision support system improves protein delivery in preterm infants: a randomized clinical trial. JPEN J Parenter Enteral Nutr. 2018;42(1):219-224. Medline:29505147 doi:10.1002/jpen.1034
25. Amaro CM, Bello JA, Jain D, et al. Early caffeine and weaning from mechanical ventilation in preterm infants: a randomized, placebo-controlled trial. J Pediatr. 2018;196:52-57. Medline:29519541 doi:10.1016/j.jpeds.2018.01.010
26. Amatya S, Macomber M, Bhutada A, Rastogi D, Rastogi S. Sudden versus gradual pressure wean from Nasal CPAP in preterm infants: a randomized controlled trial. J Perinatol. 2017;37(6):662-667. Medline:28230835 doi:10.1038/jp.2017.10
27. Anderson-Berry A, Thoene M, Wagner J, et al. Randomized trial of two doses of vitamin D3 in preterm infants <32 weeks: dose impact on achieving desired serum 25(OH)D3 in a NICU population. PLoS One. 2017;12(10):e0185950. Medline:29016653 doi:10.1371/journal.pone.0185950
28. Angeles DM, Boskovic DS, Tan JC, et al. Oral dextrose reduced procedural pain without altering cellular ATP metabolism in preterm neonates: a prospective randomized trial. J Perinatol. 2020;40(6):888-895. Medline:32103160 doi:10.1038/s41372-020-0634-0

© 2023 Lyle AJ et al. *JAMA Network Open.*

29. Angeles DM, Boskovic DS, Deming D, et al. A pilot study on the biochemical effects of repeated administration of 24% oral sucrose vs 30% oral dextrose on urinary markers of adenosine triphosphate degradation. J Perinatol. 2021;41(12):2761-2765. Medline:34671099 doi:10.1038/s41372-021-01239-8
30. Arnold C, Tyson JE, Pedroza C, et al. Cycled phototherapy dose-finding study for extremely low-birth-weight infants: a randomized clinical trial. JAMA Pediatr. 2020;174(7):649-656. Medline:32338720 doi:10.1001/jamapediatrics.2020.0559
31. Atchley CB, Cloud A, Thompson D, et al. Enhanced protein diet for preterm infants: a prospective, randomized, double-blind, controlled trial. J Pediatr Gastroenterol Nutr. 2019;69(2):218-223. Medline:31058772 doi:10.1097/MPG.0000000000002376
32. Backes CH, Cooper JN, Notestine JL, et al. A trial comparing continuous positive airway pressure (CPAP) devices in preterm infants. J Perinatol. 2020;40(8):1193-1201. Medline:32433510 doi:10.1038/s41372-020-0690-5
33. Balakrishnan M, Jennings A, Przystac L, et al. Growth and neurodevelopmental outcomes of early, high-dose parenteral amino acid intake in very low birth weight infants: a randomized controlled trial. JPEN J Parenter Enteral Nutr. 2018;42(3):597-606. Medline:29187120 doi:10.1177/0148607117696330
34. Ballengee CR, Davalian F, Conaway MR, Sauer CG, Kaufman DA. Erythromycin and reflux events in premature neonates: a randomized clinical trial. J Pediatr Gastroenterol Nutr. 2018;67(6):720-725. Medline:29985874 doi:10.1097/MPG.0000000000002086
35. Bedwell SM, Buster B, Sekar K. The effect of a continuous milk warming system on weight gain in very low birth-weight infants: a randomized controlled trial. Adv Neonatal Care. 2021;21(4):E86-E92. Medline:33427754 doi:10.1097/ANC.0000000000000818
36. Blakely ML, Tyson JE, Lally KP, et al; Eunice Kennedy Shriver National Institute of Child Health, Human Development Neonatal Research Network. Initial laparotomy versus peritoneal drainage in extremely low birthweight infants with surgical necrotizing enterocolitis or isolated intestinal perforation: a multicenter randomized clinical trial. Ann Surg. 2021;274(4):e370-e380. Medline:34506326 doi:10.1097/SLA.0000000000005099
37. Bogen DL, Hanusa BH, Baker R, Medoff-Cooper B, Cohlan B. Randomized clinical trial of standard- versus high-calorie formula for methadone-exposed infants: a feasibility study. Hosp Pediatr. 2018;8(1):7-14. Medline:29263124 doi:10.1542/hpeds.2017-0114
38. Brandon DH, Silva SG, Park J, Malcolm W, Kamhawy H, Holditch-Davis D. Timing for the introduction of cycled light for extremely preterm infants: a randomized controlled trial. Res Nurs Health. 2017;40(4):294-310. Medline:28431191 doi:10.1002/nur.21797
39. Brion LP, Rosenfeld CR, Heyne R, et al. Optimizing individual nutrition in preterm very low birth weight infants: double-blinded randomized controlled trial. J Perinatol. 2020;40(4):655-665. Medline:32071367 doi:10.1038/s41372-020-0609-1
40. Brusseau C, Burnette T, Heidel RE. Clonidine versus phenobarbital as adjunctive therapy for neonatal abstinence syndrome. J Perinatol. 2020;40(7):1050-1055. Medline:32424335 doi:10.1038/s41372-020-0685-2
41. Bruzoni M, Jaramillo JD, Dunlap JL, et al. Sutureless vs sutured gastroschisis closure: a prospective randomized controlled trial. J Am Coll Surg. 2017;224(6):1091-1096.e1. Medline:28279777 doi:10.1016/j.jamcollsurg.2017.02.014

© 2023 Lyle AJ et al. *JAMA Network Open.*

42. Butler-O’Hara M, Reininger A, Wang H, Amin SB, Rodgers NJ, D’Angio CT. A randomized controlled trial of glycerin suppositories during phototherapy in premature neonates. J Obstet Gynecol Neonatal Nurs. 2017;46(2):220-228. Medline:28082211 doi:10.1016/j.jogn.2016.10.008
43. Calkins KL, Havranek T, Kelley-Quon LI, et al. Low-dose parenteral soybean oil for the prevention of parenteral nutrition-associated liver disease in neonates with gastrointestinal disorders. JPEN J Parenter Enteral Nutr. 2017;41(3):404-411. Medline:26024828 doi:10.1177/0148607115588334
44. Ceyhan-Birsoy O, Murry JB, Machini K, et al; BabySeq Project Team. Interpretation of genomic sequencing results in healthy and ill newborns: results from the BabySeq Project. Am J Hum Genet. 2019;104(1):76-93. Medline:30609409 doi:10.1016/j.ajhg.2018.11.016
45. Choi PS, Emani S, Ibla JC, Marturano JE, Lowery TJ, Emani S. Magnetic resonance-based diagnostics for bleeding assessment in neonatal cardiac surgery. Ann Thorac Surg. 2020;109(6):1931-1936. Medline:31887277 doi:10.1016/j.athoracsur.2019.11.010
46. Cholette JM, Swartz MF, Rubenstein J, et al. Outcomes using a conservative versus liberal red blood cell transfusion strategy in infants requiring cardiac operation. Ann Thorac Surg. 2017;103(1):206-214. Medline:27496630 doi:10.1016/j.athoracsur.2016.05.049
47. Chorna OD, L Hamm E, Shrivastava H, Maitre NL. Feasibility of event-related potential (ERP) biomarker use to study effects of mother’s voice exposure on speech sound differentiation of preterm infants. Dev Neuropsychol. 2018;43(2):123-134. Medline:29436854 doi:10.1080/87565641.2018.1433671
48. Claassen CC, Hillman NH, Brown K, Williams HL, Strand ML. Comparison of bubble CPAP devices using RAM cannula for extubation failure in very low birth weight infants: randomized and cohort studies. Neonatology. 2019;115(1):28-35. Medline:30278459 doi:10.1159/000493156
49. Cook LM, Nichols-Dada J, Damani S, et al. Randomized clinical trial of 24% oral sucrose to decrease pain associated with peripheral intravenous catheter insertion in preterm and term newborns. Adv Neonatal Care. 2017;17(1):E3-E11. Medline:27533333 doi:10.1097/ANC.0000000000000326
50. Cooper DM, Girolami GL, Kepes B, et al. Body composition and neuromotor development in the year after NICU discharge in premature infants. Pediatr Res. 2020;88(3):459-465. Medline:31926484 doi:10.1038/s41390-020-0756-2
51. Corrigan MJ, Keeler JR, Miller HD, Ben Khallouq BA, Fowler SB. Music therapy and retinopathy of prematurity screening: using recorded maternal singing and heartbeat for post exam recovery. J Perinatol. 2020;40(12):1780-1788. Medline:32681063 doi:10.1038/s41372-020-0719-9
52. Cummings JJ, Gerday E, Minton S, et al; AERO-02 STUDY INVESTIGATORS. Aerosolized calfactant for newborns with respiratory distress: a randomized trial. Pediatrics. 2020;146(5):e20193967. Medline:33060258 doi:10.1542/peds.2019-3967
53. Czarnecki ML, Hainsworth K, Simpson PM, et al; Continuous Opioid Infusion in the Neonatal Intensive Care Unit. A pilot randomized controlled trial of outcomes associated with parent-nurse controlled analgesia vs continuous opioid infusion in the neonatal intensive care unit. Pain Manag Nurs. 2020;21(1):72-80. Medline:31494028 doi:10.1016/j.pmn.2019.08.002

© 2023 Lyle AJ et al. *JAMA Network Open.*

54. Czynski AJ, Davis JM, Dansereau LM, et al. Neurodevelopmental outcomes of neonates randomized to morphine or methadone for treatment of neonatal abstinence syndrome. J Pediatr. 2020;219:146-151.e1. Medline:31987653 doi:10.1016/j.jpeds.2019.12.018
55. Daniel JM, Davidson LN, Havens JR, Bauer JA, Shook LA. Aromatherapy as an adjunctive therapy for neonatal abstinence syndrome: a pilot study. J Opioid Manag. 2020;16(2):119-125. Medline:32329887 doi:10.5055/jom.2020.0558
56. Davidson J, Ruthazer R, Maron JL. Optimal timing to utilize olfactory stimulation with maternal breast milk to improve oral feeding skills in the premature newborn. Breastfeed Med. 2019;14(4):230-235. Medline:30882237 doi:10.1089/bfm.2018.0180
57. Davis JM, Shenberger J, Terrin N, et al. Comparison of safety and efficacy of methadone vs morphine for treatment of neonatal abstinence syndrome: a randomized clinical trial. JAMA Pediatr. 2018;172(8):741-748. Medline:29913015 doi:10.1001/jamapediatrics.2018.1307
58. Detmer MR, Evans K, Shina E, Walker K, DeLoach D, Malowitz JR. Multimodal neurologic enhancement improves preterm infants' developmental outcomes: a longitudinal pilot study. Neonatal Netw. 2020;39(1):16-23. Medline:31919289 doi:10.1891/0730-0832.39.1.16
59. DuPont TL, Baserga M, Lowe J, Zamora T, Beauman S, Ohls RK. Darbepoetin as a neuroprotective agent in mild neonatal encephalopathy: a randomized, placebo-controlled, feasibility trial. J Perinatol. 2021;41(6):1339-1346. Medline:33986477 doi:10.1038/s41372-021-01081-y
60. Elzein C, Urbas C, Hughes B, et al. Efficacy of nitric oxide administration in attenuating ischemia/reperfusion injury during neonatal cardiopulmonary bypass. World J Pediatr Congenit Heart Surg. 2020;11(4):417-423. Medline:32645771 doi:10.1177/2150135120911034
61. Emery L, Hamm EL, Hague K, Chorna OD, Moore-Clingenpeel M, Maitre NL. A randomised controlled trial of protocolised music therapy demonstrates developmental milestone acquisition in hospitalised infants. Acta Paediatr. 2019;108(5):828-834. Medline:30375661 doi:10.1111/apa.14628
62. Eze N, Murphy D, Dhar V, Rehan VK. Comparison of sprinting vs non-sprinting to wean nasal continuous positive airway pressure off in very preterm infants. J Perinatol. 2018;38(2):164-168. Medline:29072676 doi:10.1038/jp.2017.161
63. Foglia EE, Ades A, Hedrick HL, et al. Initiating resuscitation before umbilical cord clamping in infants with congenital diaphragmatic hernia: a pilot feasibility trial. Arch Dis Child Fetal Neonatal Ed. 2020;105(3):322-326. Medline:31462406 doi:10.1136/archdischild-2019-317477
64. Forde D, Deming DD, Tan JC, et al. Oxidative stress biomarker decreased in preterm neonates treated with kangaroo mother care. Biol Res Nurs. 2020;22(2):188-196. Medline:31973579 doi:10.1177/1099800419900231
65. Frost BL, Patel AL, Robinson DT, Berseth CL, Cooper T, Caplan M. Randomized controlled trial of early docosahexaenoic acid and arachidonic acid enteral supplementation in very low birth weight infants. J Pediatr. 2021;232:23-30.e1. Medline:33358843 doi:10.1016/j.jpeds.2020.12.037
66. Gautam NK, Pierre J, Edmonds K, et al. Transfusing platelets during bypass rewarming in neonates improves postoperative outcomes: a randomized controlled trial. World J Pediatr Congenit Heart Surg. 2020;11(1):71-76. Medline:31835978 doi:10.1177/2150135119888155

© 2023 Lyle AJ et al. *JAMA Network Open.*

67. Gaynor JW, Nicolson SC, Spray DM, et al. Remote ischemic preconditioning does not prevent white matter injury in neonates. Ann Thorac Surg. 2018;106(1):151-155. Medline:29601806 doi:10.1016/j.athoracsur.2018.02.060
68. Gerges A, Gelfer P, Kennedy K. Randomized trial of earlier versus later oral feeding in very premature infants. J Perinatol. 2018;38(6):687-692. Medline:29453433 doi:10.1038/s41372-018-0058-2
69. Glass KM, Greecher CP, Doheny KK. Oropharyngeal administration of colostrum increases salivary secretory iga levels in very low-birth-weight infants. Am J Perinatol. 2017;34(14):1389-1395. Medline:28575910 doi:10.1055/s-0037-1603655
70. Graham EM, Martin RH, Buckley JR, et al. Corticosteroid therapy in neonates undergoing cardiopulmonary bypass: randomized controlled trial. J Am Coll Cardiol. 2019;74(5):659-668. Medline:31370958 doi:10.1016/j.jacc.2019.05.060
71. Gray MM, Medoff-Cooper B, Enlow EM, Mukhopadhyay S, DeMauro SB. Every three-hour versus every six-hour oral feeding in preterm infants: a randomised clinical trial. Acta Paediatr. 2017;106(2):236-241. Medline:27862264 doi:10.1111/apa.13658
72. Gupta K, Wang H, Amin SB. Soybean-oil lipid minimization for prevention of intestinal failure-associated liver disease in late-preterm and term infants with gastrointestinal surgical disorders. JPEN J Parenter Enteral Nutr. 2021;45(6):1239-1248. Medline:32854150 doi:10.1002/jpen.2004
73. Hammer GB, Maxwell LG, Taicher BM, et al. Randomized population pharmacokinetic analysis and safety of intravenous acetaminophen for acute postoperative pain in neonates and infants. J Clin Pharmacol. 2020;60(1):16-27. Medline:31448420 doi:10.1002/jcph.1508
74. Hammond J, Kamboj R, Kashyap S, Sahni R. The interaction between diet and neurobehavior in very low birth weight infants. Pediatr Res. 2022;91(3):646-651. Medline:33767376 doi:10.1038/s41390-021-01464-z
75. Hart CK, Tawfik KO, Meinzen-Derr J, et al. A randomized controlled trial of Velcro versus standard twill ties following pediatric tracheotomy. Laryngoscope. 2017;127(9):1996-2001. Medline:28480522 doi:10.1002/lary.26608
76. Havranek T, Shatzkin E, Chuang M, Xie X, Kim M, Rosen O. Respiratory outcomes after neonatal prone versus supine positioning following scheduled cesarean delivery: a randomized trial. J Matern Fetal Neonatal Med. 2021;34(18):2938-2944. Medline:31564177 doi:10.1080/14767058.2019.1674805
77. Hibbs AM, Ross K, Kerns LA, et al. Effect of vitamin D supplementation on recurrent wheezing in Black infants who were born preterm: the D-Wheeze Randomized Clinical Trial. JAMA. 2018;319(20):2086-2094. Medline:29800180 doi:10.1001/jama.2018.5729
78. Jadcherla SR, Hasenstab KA, Wei L, et al. Role of feeding strategy bundle with acid-suppressive therapy in infants with esophageal acid reflux exposure: a randomized controlled trial. Pediatr Res. 2021;89(3):645-652. Medline:32380509 doi:10.1038/s41390-020-0932-4
79. Jakubowicz JF, Bai S, Matlock DN, et al. Effect of transcutaneous electrode temperature on accuracy and precision of carbon dioxide and oxygen measurements in the preterm infants. Respir Care. 2018;63(7):900-906. Medline:29717098 doi:10.4187/respcare.05887
80. Jooste EH, Scholl R, Wu YH, et al. Double-blind, randomized, placebo-controlled trial comparing the effects of antithrombin versus placebo on the coagulation system in infants with

© 2023 Lyle AJ et al. *JAMA Network Open.*

low antithrombin undergoing congenital cardiac surgery. J Cardiothorac Vasc Anesth. 2019;33(2):396-402. Medline:30072263 doi:10.1053/j.jvca.2018.05.052
81. Josephsen JB, Potter S, Armbrecht ES, Al-Hosni M. Umbilical cord milking in extremely preterm infants: a randomized controlled trial comparing cord milking with immediate cord clamping. Am J Perinatol. 2022;39(4):436-443. Medline:32894871 doi:10.1055/s-0040-1716484
82. Juul SE, Comstock BA, Wadhawan R, et al; PENUT Trial Consortium. A randomized trial of erythropoietin for neuroprotection in preterm infants. N Engl J Med. 2020;382(3):233-243. Medline:31940698 doi:10.1056/NEJMoa1907423
83. Kakkilaya V, Tang A, Wagner S, et al. Discontinuing nasal continuous positive airway pressure in infants ≤32 weeks of gestational age: a randomized control trial. J Pediatr. 2021;230:93-99.e3. Medline:33127365 doi:10.1016/j.jpeds.2020.10.045
84. Katheria AC, Morales A, Shashank S, Rich WD, Finer NN. A pilot randomized trial of heart rate monitoring using conventional versus a new electrocardiogram algorithm during neonatal resuscitation at birth. J Pediatr. 2022;242:245-247.e1. Medline:34715091 doi:10.1016/j.jpeds.2021.10.037
85. Katheria A, Arnell K, Brown M, et al. A pilot randomized controlled trial of EKG for neonatal resuscitation. PLoS One. 2017;12(11):e0187730. Medline:29099872 doi:10.1371/journal.pone.0187730
86. Kim CS, Grady N, Derrick M, et al. Effect of antibiotic use within first 48 hours of life on the preterm infant microbiome: a randomized clinical trial. JAMA Pediatr. 2021;175(3):303-305. Medline:33196773 doi:10.1001/jamapediatrics.2020.4916
87. Kingsmore SF, Cakici JA, Clark MM, et al; RCIGM Investigators. A randomized, controlled trial of the analytic and diagnostic performance of singleton and trio, rapid genome and exome sequencing in ill infants. Am J Hum Genet. 2019;105(4):719-733. Medline:31564432 doi:10.1016/j.ajhg.2019.08.009
88. Kirpalani H, Bell EF, Hintz SR, et al; Eunice Kennedy Shriver NICHD Neonatal Research Network. Higher or lower hemoglobin transfusion thresholds for preterm infants. N Engl J Med. 2020;383(27):2639-2651. Medline:33382931 doi:10.1056/NEJMoa2020248
89. Kochan M, Leonardi B, Firestine A, et al. Elevated midline head positioning of extremely low birth weight infants: effects on cardiopulmonary function and the incidence of periventricular-intraventricular hemorrhage. J Perinatol. 2019;39(1):54-62. Medline:30348960 doi:10.1038/s41372-018-0261-1
90. Kotloff KL, Shirley DT, Creech CB, et al. Mupirocin for Staphylococcus aureus decolonization of infants in neonatal intensive care units. Pediatrics. 2019;143(1):e20181565. Medline:30587533 doi:10.1542/peds.2018-1565
91. Kraft WK, Adeniyi-Jones SC, Chervoneva I, et al. Buprenorphine for the treatment of the neonatal abstinence syndrome. N Engl J Med. 2017;376(24):2341-2348. Medline:28468518 doi:10.1056/NEJMoa1614835
92. Kumar N, Monga R, Sampath V, Ehrhart B. Prospective comparison of Enfamil and Similac liquid human milk fortifier on clinical outcomes in premature infants. Am J Perinatol. 2017;34(14):1411-1416. Medline:28637062 doi:10.1055/s-0037-1603940
93. Lafferty MA, Mackley A, Green P, Ottenthal D, Locke R, Guillen U. Can Mozart improve weight gain and development of feeding skills in premature infants: a randomized trial. Am J Perinatol. 2023;40(7):793-798. Medline:34157772 doi:10.1055/s-0041-1731279

94. Lam R, Schilling D, Scottoline B, et al. The effect of extended continuous positive airway pressure on changes in lung volumes in stable premature infants: a randomized controlled trial. J Pediatr. 2020;217:66-72.e1. Medline:31519441 doi:10.1016/j.jpeds.2019.07.074
95. Lambert LM, Trachtenberg FL, Pemberton VL, et al; Pediatric Heart Network Investigators. Passive range of motion exercise to enhance growth in infants following the Norwood procedure: a safety and feasibility trial. Cardiol Young. 2017;27(7):1361-1368. Medline:28330522 doi:10.1017/S1047951117000427
96. Laptook AR, Shankaran S, Tyson JE, et al; Eunice Kennedy Shriver National Institute of Child Health and Human Development Neonatal Research Network. Effect of therapeutic hypothermia initiated after 6 hours of age on death or disability among newborns with hypoxic-ischemic encephalopathy: a randomized clinical trial. JAMA. 2017;318(16):1550-1560. Medline:29067428 doi:10.1001/jama.2017.14972
97. Makker K, Cortez J, Jha K, et al. Comparison of extubation success using noninvasive positive pressure ventilation (NIPPV) versus noninvasive neurally adjusted ventilatory assist (NI-NAVA). J Perinatol. 2020;40(8):1202-1210. Medline:31911641 doi:10.1038/s41372-019-0578-4
98. Marr BL, Mettelman BB, Bode MM, Gross SJ. Randomized trial of 42-day compared with 9-day courses of dexamethasone for the treatment of evolving bronchopulmonary dysplasia in extremely preterm infants. J Pediatr. 2019;211:20-26.e1. Medline:31349916 doi:10.1016/j.jpeds.2019.04.047
99. McMichael ABV, Zimmerman KO, Kumar KR, Ozment CP. Evaluation of effect of scheduled fresh frozen plasma on ECMO circuit life: a randomized pilot trial. Transfusion. 2021;61(1):42-51. Medline:33269487 doi:10.1111/trf.16164
100. Mu TS, Prescott AC, Haischer-Rollo GD, Aden JK, Shapiro JB. Umbilical cord blood use for admission blood tests of VLBW preterm neonates: a randomized control trial. Am J Perinatol. 2023;40(10):1119-1125. Medline:34407547 doi:10.1542/peds.147.3_MeetingAbstract.1058
101. Napolitano N, Dysart K, Soorikian L, Zhang H, Panitch H, Jensen E. Tolerability and efficacy of two doses of aerosolized albuterol in ventilated infants with BPD: a randomized controlled crossover trial. Pediatr Pulmonol. 2021;56(1):97-104. Medline:33095509 doi:10.1002/ppul.25131
102. Nelson MU, Shaw J, Gross SJ. Randomized placebo-controlled trial of topical mupirocin to reduce staphylococcus aureus colonization in infants in the neonatal intensive care unit. J Pediatr. 2021;236:70-77. Medline:34023342 doi:10.1016/j.jpeds.2021.05.042
103. Niebler RA, Chiang-Ching H, Daley K, et al. Nitric oxide added to the sweep gas of the oxygenator during cardiopulmonary bypass in infants: a pilot randomized controlled trial. Artif Organs. 2021;45(1):22-28. Medline:32737900 doi:10.1111/aor.13788
104. Pandey R, Kanike N, Ibrahim M, et al. Lactose-free infant formula does not change outcomes of neonatal abstinence syndrome (NAS): a randomized clinical trial. J Perinatol. 2021;41(3):598-605. Medline:32868858 doi:10.1038/s41372-020-00797-7
105. Parker LA, Weaver M, Murgas Torrazza RJ, et al. Effect of gastric residual evaluation on enteral intake in extremely preterm infants: a randomized clinical trial. JAMA Pediatr. 2019;173(6):534-543. Medline:31034045 doi:10.1001/jamapediatrics.2019.0800
106. Perretta LJ, Spaight M, Yap V, Perlman J. Randomized study of delayed cord clamping of 30 to 60 seconds in the larger infant born preterm. J Pediatr. 2020;224:153-157. Medline:32651013 doi:10.1016/j.jpeds.2020.04.058

© 2023 Lyle AJ et al. *JAMA Network Open.*

107. Petrikin JE, Cakici JA, Clark MM, et al. The NSIGHT1-randomized controlled trial: rapid whole-genome sequencing for accelerated etiologic diagnosis in critically ill infants. NPJ Genom Med. 2018;3:6. Medline:29449963 doi:10.1038/s41525-018-0045-8
108. Phelps DL, Watterberg KL, Nolen TL, et al; Eunice Kennedy Shriver National Institute of Child Health and Human Development Neonatal Research Network. Effects of myo-inositol on type 1 retinopathy of prematurity among preterm infants <28 weeks' gestational age: a randomized clinical trial. JAMA. 2018;320(16):1649-1658. Medline:30357297 doi:10.1001/jama.2018.14996
109. Pickler RH, Meinzen-Derr J, Moore M, Sealschott S, Tepe K. Effect of Tactile Experience During Preterm Infant Feeding on Clinical Outcomes. Nurs Res. 2020;69(5S)(suppl 1):S21-S28. Medline:32555011 doi:10.1097/NNR.0000000000000453
110. Pineda R, Smith J, Roussin J, Wallendorf M, Kellner P, Colditz G. Randomized clinical trial investigating the effect of consistent, developmentally-appropriate, and evidence-based multisensory exposures in the NICU. J Perinatol. 2021;41(10):2449-2462. Medline:34012055 doi:10.1038/s41372-021-01078-7
111. Poola AS, Aguayo P, Fraser JD, et al. Primary closure versus bedside silo and delayed closure for gastroschisis: a truncated prospective randomized trial. Eur J Pediatr Surg. 2019;29(2):203-208. Medline:29458229 doi:10.1055/s-0038-1627459
112. Pourmoghadam KK, Kubovec S, DeCampli WM, et al. Passive peritoneal drainage impact on fluid balance and inflammatory mediators: a randomized pilot study. World J Pediatr Congenit Heart Surg. 2020;11(2):150-158. Medline:32093557 doi:10.1177/2150135119888143
113. Ramanathan R, Biniwale M, Sekar K, et al. Synthetic surfactant CHF5633 Compared with poractant alfa in the Treatment of neonatal respiratory distress syndrome: a multicenter, double-blind, randomized, controlled clinical trial. J Pediatr. 2020;225:90-96.e1. Medline:32553868 doi:10.1016/j.jpeds.2020.06.024
114. Roberts KD, Brown R, Lampland AL, et al. Laryngeal mask airway for surfactant administration in neonates: a randomized, controlled trial. J Pediatr. 2018;193:40-46.e1. Medline:29174079 doi:10.1016/j.jpeds.2017.09.068
115. Romano-Keeler J, Azcarate-Peril MA, Weitkamp JH, et al. Oral colostrum priming shortens hospitalization without changing the immunomicrobial milieu. J Perinatol. 2017;37(1):36-41. Medline:27684425 doi:10.1038/jp.2016.161
116. Rosenfeld WN, Hudak ML, Ruiz N, Gautam S; Jasmine Study Group. Stannsoporfin with phototherapy to treat hyperbilirubinemia in newborn hemolytic disease. J Perinatol. 2022;42(1):110-115. Medline:34635771 doi:10.1038/s41372-021-01223-2
117. Rosterman JL, Pallotto EK, Truog WE, et al. The impact of neurally adjusted ventilatory assist mode on respiratory severity score and energy expenditure in infants: a randomized crossover trial. J Perinatol. 2018;38(1):59-63. Medline:29072677 doi:10.1038/jp.2017.154
118. Ruoss JL, Bazacliu C, Russell JT, et al. Routine early antibiotic use in symptomatic preterm neonates: a pilot randomized controlled trial. J Pediatr. 2021;229:294-298.e3. Medline:32979383 doi:10.1016/j.jpeds.2020.09.056
119. Salas AA, Jerome ML, Chandler-Laney P, Ambalavanan N, Carlo WA. Serial assessment of fat and fat-free mass accretion in very preterm infants: a randomized trial. Pediatr Res. 2020;88(5):733-738. Medline:32634820 doi:10.1038/s41390-020-1052-x

© 2023 Lyle AJ et al. *JAMA Network Open.*

120. Salas AA, Jerome M, Finck A, Razzaghy J, Chandler-Laney P, Carlo WA. Body composition of extremely preterm infants fed protein-enriched, fortified milk: a randomized trial. Pediatr Res. 2022;91(5):1231-1237. Medline:34183770 doi:10.1038/s41390-021-01628-x
121. Salas AA, Li P, Parks K, Lal CV, Martin CR, Carlo WA. Early progressive feeding in extremely preterm infants: a randomized trial. Am J Clin Nutr. 2018;107(3):365-370. Medline:29529231 doi:10.1093/ajcn/nqy012
122. Salas AA, Woodfin T, Phillips V, Peralta-Carcelen M, Carlo WA, Ambalavanan N. Dose-response effects of early vitamin D supplementation on neurodevelopmental and respiratory outcomes of extremely preterm infants at 2 years of age: a randomized trial. Neonatology. 2018;113(3):256-262. Medline:29393233 doi:10.1159/000484399
123. Schanler RJ, Groh-Wargo SL, Barrett-Reis B, et al. Improved outcomes in preterm infants fed a nonacidified liquid human milk fortifier: a prospective randomized clinical trial. J Pediatr. 2018;202:31-37.e2. Medline:30195561 doi:10.1016/j.jpeds.2018.07.005
124. Sekar K, Szyld E, McCoy M, et al. Inhaled nitric oxide as an adjunct to neonatal resuscitation in premature infants: a pilot, double blind, randomized controlled trial. Pediatr Res. 2020;87(3):523-528. Medline:31666688 doi:10.1038/s41390-019-0643-x
125. Shankaran S, Laptook AR, Pappas A, et al; Eunice Kennedy Shriver National Institute of Child Health and Human Development Neonatal Research Network. Effect of depth and duration of cooling on death or disability at age 18 months among neonates with hypoxic-ischemic encephalopathy: a randomized clinical trial. JAMA. 2017;318(1):57-67. Medline:28672318 doi:10.1001/jama.2017.7218
126. Shankaran S, Bell EF, Laptook AR, et al; Eunice Kennedy Shriver National Institute of Child Health, and Human Development Neonatal Research Network. Weaning of moderately preterm infants from the incubator to the crib: a randomized clinical trial. J Pediatr. 2019;204:96-102.e4. Medline:30337189 doi:10.1016/j.jpeds.2018.08.079
127. Shellhaas RA, Burns JW, Barks JDE, Hassan F, Chervin RD. Maternal voice and infant sleep in the neonatal intensive care unit. Pediatrics. 2019;144(3):e20190288. Medline:31409691 doi:10.1542/peds.2019-0288
128. Shirk SK, Manolis SA, Lambers DS, Smith KL. Delayed clamping vs milking of umbilical cord in preterm infants: a randomized controlled trial. Am J Obstet Gynecol. 2019;220(5):482.e1-482.e8. Medline:30786254 doi:10.1016/j.ajog.2019.01.234
129. Smith S, Keltner C, Stikes R, Hay

© 2023 Lyle AJ et al. *JAMA Network Open.*
