# Supplementary material for: Race and Ethnicity of Infants Enrolled in Neonatal Clinical Trials: A Systematic Review
Source: JAMA Netw Open. 2023 Dec 21;6(12):e2348882. doi: 10.1001/jamanetworkopen.2023.48882 (PMC10739112; doi:10.1001/jamanetworkopen.2023.48882)
Supplement: Supplement 2. — Data Sharing Statement [file jamanetwopen-e2348882-s002.pdf]

Data Sharing Statement

Lyle. Race and Ethnicity of Infants Enrolled in Neonatal Clinical Trials. *JAMA Netw Open*.
Published December 21, 2023. doi:10.1001/jamanetworkopen.2023.48882

Data
Data available: No

Additional Information
Explanation for why data not available: no new data created
